# Fruiting character variability in wild individuals of *Malania oleifera*, a highly valued endemic species

**DOI:** 10.1038/s41598-021-03080-7

**Published:** 2021-12-08

**Authors:** Si-Hai Wang, Jian Chen, Wei Yang, Mei Hua, Yong-Peng Ma

**Affiliations:** 1 Yunnan Provincial Key Laboratory of Forest Plant Cultivation and Utilization, Yunnan Academy of Forestry and Grassland, Kunming, 650201 China; 2 Key Laboratory of the State Forestry Administration on Conservation of Rare, Endangered and Endemic Forest Plants, Kunming, 650201 China; 3 Yunnan Key Laboratory for Integrative Conservation of Plant Species With Extremely Small Populations, Kunming Institute of Botany, Chinese Academy of Sciences, Kunming, 650201 China

**Keywords:** Ecology, Plant sciences

## Abstract

*Malania oleifera* (Olacaceae), a tree species endemic to Southwest China, has seed oils enriched with nervonic acid and is therefore good source of this chemical. Because of this, there are promising industrial perspective in the artificial cultivation and use of this species. Understanding the variability in the fruit characters among individuals forms the basis or resource prospection. In the current investigation, fifty-three mature fruiting trees were sampled from two locations with divergent climates (Guangnan and Funing). Morphological characterization of fruits (fruit and stone weight, fruit transverse and longitudinal diameter, stone transverse and longitudinal diameter) was conducted, and the concentration of seed oil and its fatty acid composition were also analyzed in all individuals. Differences in all the morphological characters studied were more significant among individual trees than between different geographic localities, even though these had different climates. Eleven fatty acids were identified contributing between 91.39 and 96.34% of the lipids, and the major components were nervonic acid (38.93–47.24%), octadecenoic acid (26.79–32.08%), docosenoic acid (10.94–17.24%). The seed oil content (proportion of oil in seed kernel) and the proportion of nervonic acid were both higher in Funing, which has a higher average climatic temperature than Guangnan. The concentrations of nervonic acid and octadecenoic acid with the low coefficients of variation in the seed oil of *M. oleifera* were relatively stable in contrast to the other fatty acids. There were significant positive correlations between fruit morphological characters, but the amount of seed oil and the concentrations of its components were not correlated with any morphological character. This study provides an understanding of morphological variation in wild *M. oleifera* individuals. Wild individuals with excellent fruit traits could be selected and would make promising candidates for commercial cultivation.

Biological resources, especially highly valued endemic species, are important strategic resources for the sustainable development of local economies and societies across the world^1,2^. Prospecting and utilizing novel resources from plants has become an efficient way to promote the sustainable development of human societies, and can also be effective way to provide new solutions to old problems including drug discovery, health promotion, food security, or finding new energy sources^3,4^. Among the plant species used as resources, endemic plants often have unique value and play important or irreplaceable roles in local communities^5–7^. China has one of the highest levels of biodiversity in the world, and has very high levels of plant endemism^8^. These endemic plant species have great potential as novel bio-resources.

*Malania oleifera* Chun & S.K. Lee, a monotypic species belonging to the Olacaceae family, is an endemic and endangered tree species, and is only found naturally scattered through karst landscapes of southeastern Yunnan province and western Guangxi province, China^9,10^. *M. oleifera* is listed in the IUCN Red List as VU (vulnerable)^11^, is recorded in the “Red Data Book of Chinese Plants: Rare and Endangered Plants”^12^, and was recently assigned as a plant species with extremely small population sizes with high conservation priority^13^. The karst regions of Southwest China, in which *M. oleifera* is found naturally, are regions that are economically under-developed. Up to the 1980s, *M. oleifera* was known only by a few local communities, where the seeds were used to make a poor-tasting edible oil. The species was described scientifically in 1980^14^. In 1981, it was found that the seed oil from *M. oleifera* was rich in nervonic acid (15-tetracosenic acid), and the potentially high economic value of the species was recognized for the first time^15^. Nervonic acid can make up to 55.7–67.0% of the total fatty acids of *M. oleifera* seed oil^15,16^, so the tree species is a good source of nervonic acid. The nutrient plays a vital role in human health and has significant biological functions, including the development and maintenance of the brain, improving memory, and delaying brain aging^17^. *M. oleifera* is a good candidate for the discovery and development of nervonic acid resources because of its seed oils, which are enriched in this chemical^18^. However, there are only a very few wild *M. oleifera* individuals remaining, and many of the trees fruit only rarely, so the total fruit output is extremely limited and insufficient for industrial utilization^19^. In natural communities of *M. oleifera*, seedlings and saplings are scarce, and population regeneration is difficult as a result of both human and natural disturbances^10^. Moreover, in recent years, there has been illegal and unregulated collection of the fruits of *M. oleifera*, which has caused further destruction of the wild resources and has endangered this wild plant more seriously^19^. Artificial cultivation through domestication to replace wild resources would be an effective approach both for the conservation and the sustainable utilization of *M. oleifera*, as well as for the commercial use of this species.

The fruit of *M. oleifera* comprises the outer pericarp (exocarp and mesocarp), the endocarp and the seed^16^. The stone includes both the endocarp and the seed. The mean weights and dimensions of fresh fruits and stones have been found to be different in different sites^19^^.^^20^. Seed oil content was found to represent between 51.8 and 64.5% from different literature reports^15,16,21–23^. More than 10 fatty acids have been identified from the seed oil, of which nervonic acid represented the largest fraction^15,16,21,22^. Furthermore, Yang et al.^18^ assembled and characterized the transcriptome of *M. oleifera* seeds at two developmental stages (the initial and fast oil accumulation stages), and explored the physiological and molecular mechanisms of nervonic acid biosynthesis and oil accumulation in *M. oleifera* seeds. Xu et al.^24^ provided the genome assembly and gene annotation for *M. oleifera*. However, for artificial selection and cultivation of successful varieties, the fruiting characteristics of wild individual trees are necessary, and the character variability in *M. oleifera* individuals remains unknown.

In this study, our aims are (a) to clarify the differences in fruit weight and dimensions, seed oil content, fatty acid component content among individual trees, (b) to investigate fruiting variability between two locations with divergent climates, (c) to analyze the correlations among morphological traits, and d), to understand the diversity of fruiting traits in naturally growing *M. oleifera*.

## Materials and methods

### Plant materials

Samples were collected from Guangnan and Funing counties (Yunnan province, China) from trees at the fruit ripening stage, from the end of September to early October, 2018. The voucher specimen, S.H. Wang 1809301 (YAF00051456, YCP00026587), was identified as *M. oleifera* Chun & S.K. Lee by Dr. Y. K. Sima and its sheets are stored at the herbaria, YAF and YCP (The Herbarium Codes can be searched on the website: http://sweetgum.nybg.org/ih/herbarium.php). The collection of all samples completely complies with local and national legislation permission, and permission was obtained for collection of plant. These two sampling locations represent the highest and lowest altitudes (300 and 1400 m) in the natural range of *M. oleifera*, and have different climatic characteristics^10,19^ (see Table 1, below). Table 1 shows the main geographical and climate features of the two sites. Fruiting trees were selected from both locations, where the most of individuals were scattered at each population, and sometimes several individuals grew together in a relatively close distance (tens of meters). After filtering out the individuals that did not bear fruits, bore little fruits, and were too close to each other, 28 and 25 individual trees were sampled in Guangnan and Funing, respectively. The diameter at breast height (DBH) of the sampled individuals ranged from 19 to 55 cm. Fifty fruits were harvested randomly from each tree. The weight, transverse diameter, and longitudinal diameter of the whole fruits and the stones were measured for each of the fresh fruits using an electronic balance (0.01 g precision) or vernier calipers (0.01 mm precision). After that, the seeds used for extraction of seed oil (from which the hard endocarps were not removed until use) were air-dried and stored in the shade at ambient temperature until needed^16^.

**Table 1:** Geographical description for the collection sites of *Malania oleifera*.

Location	Altitude (m)	Co-ordinates	Annual precipitation (mm)	Mean annual temperature (°C)	Mean temperature (July) (°C)	Mean temperature (January) (°C)
GN	1089–1345	23°56′ N 104°53′ E	1057	16.7	22.5	8.1
FN	352–685	23°44′ N 106°05′ E	1184	20.3	26.2	11.7

*GN* Guangnan, *FN* Funing.

### Seed oil extraction

Thirty seeds per tree were randomly selected from the fruits collected above. The seeds, together with the hard endocarps, were extracted from the fruits, air-dried and stored in the shade until needed. Shortly before oil extraction, the endocarps were removed using a nutcracker, and the seeds from each individual tree were ground together into paste using a mortar and pestle. 10 g of seed paste was taken into a 100 mL centrifuge tube and mixed with 40 mL petroleum ether (60–90 °C), then the mixture was stirred well and treated ultrasonically for 40 min. The liquid mixture was centrifuged for 15 min at 5000 rpm, after which the clear supernatant was removed and collected in a rotary evaporator flask. Each sample was re-suspended in 40 mL petroleum ether and the extraction process was repeated a total four times. The combined supernatant from four extractions was subjected to evaporation under reduced pressure in a rotary evaporator and the remaining seed oil was weighed. The assessment of the oil content of the seed paste was repeated three times from each individual tree.

### Methylation of fatty acids

The total fatty acids from the seed oil were transformed into their corresponding methyl esters. A 6% solution of potassium hydroxide in ethanol was mixed with 5 g seed oil for saponification via refluxing and heating, and following this, 250 mg of saponified fatty acids were esterified with 0.5 mL of sulfuric acid in methyl alcohol^25^.

### Gas chromatography-mass spectrometry (GC-MS) and identification of compounds

Fatty acid methyl esters were analyzed using GC-MS (6890GC/5973MS, Agilent Technologies, USA) with electron ionization (70 eV), equipped with a HP-5MS fused silica capillary column (30 mm × 0.25 mm × 0.25 μm). The initial column temperature was held at 150 °C, then raised to 280 °C at rate of 3 °C/min and held for 10 min. The carrier gas was helium, and had a flow rate of 1.2 mL/min, the pressure was 100 kPa, and the split ratio was 10:1. Fatty acid methyl esters were identified by comparison of their retention times with those of pure reference standards. Quantitative data were obtained from the electronic integration of the FID peak areas.

### Statistical analyses

The fruit weight (FW), stone weight (SW), fruit transverse diameter (FTD), fruit longitudinal diameter (FLD), stone transverse diameter (STD), stone longitudinal diameter (SLD), outer pericarp thickness (OPT), seed oil content (SOC) and fatty acid composition were used for data analysis and to draw conclusions regarding individual trees. The outer pericarp thickness (OPT) was calculated by the following formula: *OPT* = [(*FTD* − *STD*)/2 + (*FLD* − *SLD*)/2]/2. The minimum, maximum and mean value, the standard deviation (SD), and the coefficient of variation (CV) were calculated for the measured traits among individuals in Guangnan and Funing, separately. To test whether the measured traits differed significantly between Guangnan and Funing, we ran independent-samples T-tests. One-way analysis of variance (ANOVA) was used to determine significant differences in the measured traits among individuals at Guangnan and Funing. Pearson correlation coefficients were then used to determine relationships between the traits. The data were analyzed using the SPSS v16.0 statistical software (SPSS Inc., Chicago, IL, USA). The figures were generated using the software program R (version 4.1.0).

## Results and discussion

### Weight and dimensions of fruit and stone

The mean weight of a fruit from a particular tree ranged from 21.25 ± 4.26 to 58.26 ± 10.44 g, with the weight of the heaviest mean fruit weight being 2.74 times that of the lightest. Similarly, the mean stone weight ranged from 8.99 ± 2.35 to 20.32 ± 3.14 g, with a 2.26 times difference between the heaviest and lightest stones (Table 2). There were significant differences (*p* < 0.001) in the mean weights of fruits and stones among individuals, whether in Guannan or Funing (see Supplementary Information Figure S1, Table S1 and Table S4), and there was also a significant difference (*p* < 0.05) in the mean fruit weight of individuals from Guangnan and Funing, but mean stone weight was not found to be significantly different (*p* < 0.05) between the two sites (Table 2). The fruit weight, stone weight and outer pericarp thickness showed higher CVs both in Guangnan (15.59%, 11.82% and 13.92%, respectively) and Funing (25.27%, 19.47% and 21.25%, respectively) than the other traits (Table 2). Lv et al.^20^ reported a mean fruit weight of 21.30 ± 4.25 g and mean stone weight of 9.35 ± 1.68 g from a mixed sample of five *M. oleifera* individuals from Leye county, Guangxi. Guo et al.^19^ collected the fruits from various sites in Guangnan county, Yunnan, and mean fruit weights of individuals from each site ranged from 35.77 ± 7.58 to 47.29 ± 9.55 g, and mean stone weights from 14.03 ± 2.82 to 18.77 ± 3.26 g.

**Table 2:** Fruit characteristics of *Malania oleifera* individuals in Guangnan and Funing (mean ± SD).

Trait	Guangnan				Funing				*p* values
	Min	Max	Mean	CV (%)	Min	Max	Mean	CV (%)	
FW (g)	30.98 ± 670	56.38 ± 8.30	39.70 ± 6.19	15.59	21.25 ± 4.26	58.26 ± 10.44	34.90 ± 8.82	25.27	*p* < 0.05
SW (g)	12.26 ± 1.84	20.20 ± 3.17	15.48 ± 1.83	11.82	8.99 ± 2.35	20.32 ± 3.14	14.38 ± 2.80	19.47	*p* > 0.05
FTD (mm)	39.14 ± 2.72	47.89 ± 2.60	41.99 ± 2.25	5.36	32.59 ± 3.91	48.95 ± 3.04	40.21 ± 3.93	9.77	*p* > 0.05
FLD (mm)	33.61 ± 2.41	43.30 ± 2.23	37.50 ± 2.38	6.35	31.57 ± 3.83	44.99 ± 3.22	36.22 ± 3.44	9.50	*p* > 0.05
STD (mm)	29.58 ± 1.57	35.36 ± 2.66	32.08 ± 1.31	4.08	25.46 ± 3.85	35.13 ± 1.99	30.86 ± 2.37	7.68	*p* < 0.05
SLD (mm)	24.14 ± 1.71	30.63 ± 2.25	27.04 ± 1.66	6.14	22.12 ± 4.00	32.91 ± 1.94	26.52 ± 2.81	10.60	*p* > 0.05
OPT (mm)	3.71 ± 0.52	6.67 ± 0.82	5.10 ± 0.71	13.92	2.73 ± 0.58	6.79 ± 1.07	4.80 ± 1.02	21.25	*p* > 0.05
SOC (%)	48.31 ± 0.47	64.53 ± 2.33	58.15 ± 3.44	5.92	58.60 ± 2.60	67.93 ± 1.07	64.16 ± 2.93	4.57	*p* < 0.01

*FW* mean fruit weight, *SW* mean stone weight, *FTD* mean fruit transverse diameter, *FLD* mean fruit longitudinal diameter, *STD* mean stone transverse diameter, *SLD* mean stone longitudinal diameter, *OPT* outer pericarp thickness, *SOC* seed oil content, *Max* maximum value in individual trees, *Min* minimum value in individual trees, *Mean* mean value among individual trees at one site.

*p* values indicate significantly different level between Guangnan and Funing.

**Figure 1:**
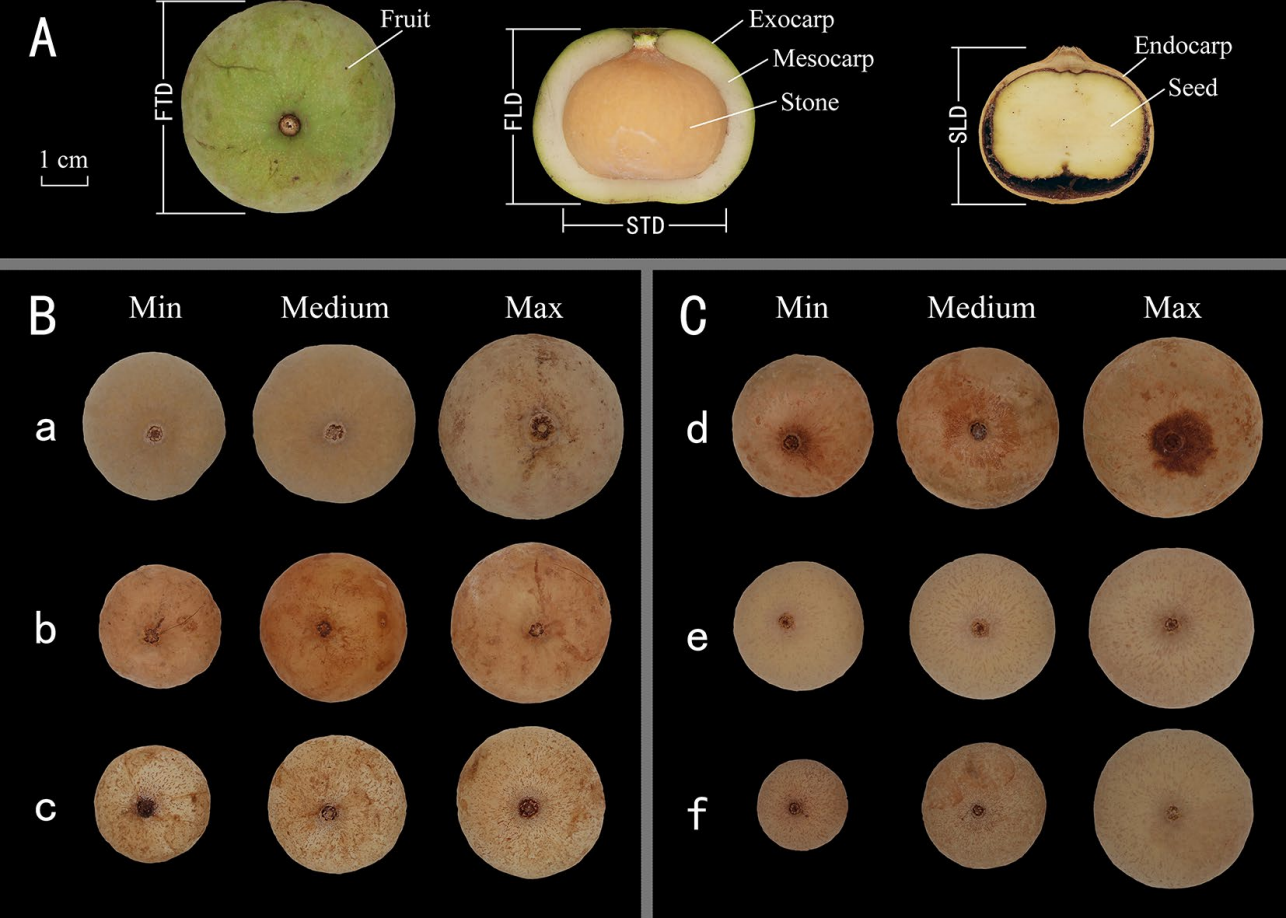
Fruit morphology of *Malania oleifera*. (**A**) Structure of fruit. (**B**) Stone size comparison of samples from Guangnan. (**C**) Stone size comparison of samples from Funing. (a) and (d): individuals with the largest average STD. (b) and (e): individuals with the medium average STD. (c) and (f): individuals with the smallest average STD. *FTD* fruit transverse diameter, *FLD* fruit longitudinal diameter, *STD* stone transverse diameter, *SLD* stone longitudinal diameter.

The mean transverse and longitudinal diameters of fruit were from 32.59 ± 3.91 to 48.95 ± 3.04 mm and 31.57 ± 3.83 to 44.99 ± 3.22 mm, respectively. The transverse and longitudinal diameters of the stone ranged from 25.46 ± 3.85 to 35.36 ± 2.66 mm and 22.12 ± 4.00 to 32.91 ± 1.94 mm, respectively. The thickness of the outer pericarp ranged from 2.73 ± 0.58 to 6.79 ± 1.07 mm (Table 2, Fig. 1). The differences in the transverse and longitudinal diameters of both fruits and stones among individuals were significant (*p* < 0.001) at both Guangnan and Funing (see Supplementary Information Figure S2, Table S2, Table S3, Table S5, Table S6). None of the fruit and stone dimensions were not significantly different (*p* > 0.05) between Guangnan and Funing, except for the stone transverse diameter (*p* < 0.05). The CVs in Funing for the traits FTD (9.77%), FLD (9.50%), STD (7.68%), SLD (10.60%), and OPT (21.25%) were higher than those in Guangnan (5.36%, 6.35%, 4.08%, 6.14%, and 13.92%, respectively) (Table 2).

The ranges of fruit transverse and longitudinal diameters have been previously reported to be from 39.31 ± 2.77 to 45.03 ± 3.47 mm and 35.19 ± 2.25 to 40.36 ± 3.49 mm, respectively, and the stone transverse and longitudinal diameters have been reported to range from 28.61 ± 1.55 to 34.16 ± 2.21 mm and 26.12 ± 1.80 to 31.69 ± 2.33 mm, respectively, mixed tree samples^19,20^. In these studies, the fruit characters were measured according to mixed sampling from some trees, but the differences among individual trees was under-examined. Characteristics analysis of individual trees is more conducive to the selection of excellent traits for domestication. The assessment of a tree species’ phenotypic variation is a key starting point in any domestication program^26^. Some reports indicated plant species usually exhibit distinct difference in fruiting characteristics both within and amongst populations^27–29^. The variations could possibly be related to either genetic or environmental factors^26^. To a large extent, the flowering and fruiting of trees in their natural habitats are dominated by the environment^30^. However, the comparison of trait selections in the same environment allowed better evaluation of genotype influence on plant characteristics^31^. Our study suggests that morphological characters were more different among different individual trees than between different geographic localities, even though these had different climates. This means that the different genotypes of the individuals we tested may have largely contributed to the differences in phenotypes between individuals. Consequently, our analysis of morphological characters from individual trees, has allowed us to screen for some excellent individuals with the leading fruiting traits (heavier weight and larger size for fruits and stones). Individuals revealed by this study to have significantly better fruiting traits than the others include GN11 and FN18 (see Supplementary Information Figure S1, Figure S2, Table S1–S6).

### Seed oil content and fatty acid composition

The ANOVA revealed significant differences (*p* < 0.001) in seed oil content among individuals at both in Guangnan and Funing (see Supplementary Information Table S7, Table S8). The percentage of seed oil renged from 48.31 ± 0.47% to 67.93 ± 1.07% (Table 2). The CVs were 5.92% and 4.57% in Guangnan and Fujing, respectively. It is worth noting that the seed oil content in individuals was significantly different (*p* < 0.01) between Guangnan (58.15 ± 3.44%) and Funing (64.16 ± 2.93%). Previous studies have suggested that seed oil content in *M. oleifera* was about 60.25% ± 2.5%^16^, 64.5%^15^, 51.3% and 52.7%^21^, and 51.7% and 56.8%^22^. However, these previous studies used a mixed seed sample from trees and did not investigate the differences in seed oil concentration among individual trees or between sites with divergent climates.

Eleven compounds were identified from the analysis of the fatty acids in the seed oil. Together, these eleven compounds contributed between 91.39 to 96.34% of the total lipids. The most abundant fatty acid was nervonic acid (38.93–47.24%), and the other major fatty acids were octadecenoic acid (26.79–32.08%), docosenoic acid (10.94–17.24%), dodecenoic acid (1.98–3.11%), tetracosanoic acid (1.39–2.61%), octadecadienoic acid (0.66–1.79%), docosanoic acid (0.90–1.37%), hexadecanoic acid (0.50–0.77%), octadecanoic acid (0.31–0.58%), arachidic acid (0.22–0.32%), and hexadecenoic acid (0.06–0.15%) (Table 3). Trees in Funing had significantly higher nervonic acid content in their seed oils than did those at Guangnan (*p* < 0.01), but the docosenoic acid content in the seed oil was significantly higher in trees at Guangnan (*p* < 0.01) than those at Funing, and the concentration of octadecenoic acid in the seed oil did not differ significantly between the two sites (*p* > 0.05) (Table 3). That these 11 fatty acids are the most prevalent in *M. oleifera* seed is largely consistent with the findings of other studies. Tang et al.^16^ identified ten of eleven fatty acids above in seed oil, but did not find hexadecenoic acid. The largest fraction was represented by nervonic acid (55.70%), followed by octadecenoic acid (23.81%), docosenoic acid (13.13%), tetracosanoic acid (2.65%) and dodecenoic acid (1.28%). Peng et al.^32^ identified the same ten fatty acids as Tang et al.^16^, with the major components being nervonic acid (33.17 ± 2.75%), octadecenoic acid (27.82 ± 2.04%), docosenoic acid (12.90 ± 0.96%), arachidic acid (3.91 ± 1.28%), dodecenoic acid (3.04 ± 0.70%), and tetracosanoic acid (2.78 ± 0.37%). Cao et al.^23^ reported ten fatty acids in *M. oleifera* seed oil, but in this case the analysis identified linolenic acid (0.55%), but neither octadecanoic acid nor arachidic acid, and the fatty acid at the highest concentration was octadecenoic acid (37.88%) rather than nervonic acid (32.15%). However, across all of these studied, the three fatty acids present in the largest amounts were nervonic acid, octadecenoic acid and docosenoic acid, which together made up more than 73% (to as much as 92%) of the total lipids. Ou^15^ reported that the nervonic acid content alone could reach up to 67% of the total lipids.

**Table 3:** Fatty acid composition of seed oil from seeds taken from individual trees in Guangnan and Funing (as methyl esters).

Fatty acid	Guangnan				Funing				*p* values
	Max (%)	Min (%)	Mean (%)	CV (%)	Max (%)	Min (%)	Mean (%)	CV (%)	
HEA	0.15	0.06	0.11 ± 0.02	18.18	0.15	0.10	0.13 ± 0.02	15.38	*p* < 0.01
HAA	0.77	0.58	0.66 ± 0.06	9.09	0.75	0.50	0.62 ± 0.06	9.68	*p* < 0.05
ODA	1.79	0.92	1.33 ± 0.20	15.04	1.55	0.66	1.13 ± 0.22	19.47	*p* < 0.01
OEA	32.08	26.79	29.0 3 ± 1.28	4.41	31.23	28.35	29.28 ± 1.28	4.37	*p* > 0.05
OAA	0.56	0.31	0.41 ± 0.06	14.63	0.58	0.35	0.45 ± 0.07	15.56	*p* > 0.05
DEA	3.11	2.30	2.61 ± 0.24	9.20	3.06	1.98	2.41 ± 0.27	11.20	*p* < 0.01
ACC	0.32	0.27	0.29 ± 0.02	6.90	0.31	0.22	0.26 ± 0.03	11.54	*p* < 0.01
DSA	17.24	13.04	14.67 ± 1.11	7.57	14.88	10.94	12.59 ± 1.02	8.10	*p* < 0.01
DAA	1.37	1.10	1.23 ± 0.08	6.50	1.29	0.90	1.05 ± 0.11	10.48	*p* < 0.01
NVA	45.25	38.93	43.01 ± 1.70	3.95	47.24	43.10	44.95 ± 1.39	3.09	*p* < 0.01
TAA	2.58	1.39	1.97 ± 0.32	16.24	2.61	1.69	2.13 ± 0.28	13.15	*p* > 0.05

*HEA* hexadecenoic acid, *HAA* hexadecanoic acid, *ODA* octadecadienoic acid, *OEA* octadecenoic acid, *OAA* octadecanoic acid, *DEA* dodecenoic acid, *ACC* arachidic acid, *DSA* docosenoic acid, *DAA* docosanoic acid, *NVA* nervonic acid, *TAA* tetracosanoic acid.

*p* values indicate levels of significance in the differences between Guangnan and Funing.

Of all the fatty acids, nervonic acid had the lowest CVs (3.95% and 3.09% at Guangnan and Funing , respectively), followed by octadecenoic acid (4.41% and 4.37%), and docosenoic acid (7.57% and 8.10%), docosanoic acid (6.50% and 10.48%), arachidic acid (6.90% and 11.54%), hexadecanoic acid (9.09% and 9.68%), dodecenoic acid (9.20% and 11.20%), tetracosanoic acid (16.24% and 13.15%), octadecanoic acid (14.63% and 15.56%), hexadecenoic acid (18.18% and 15.38%), and octadecadienoic acid (15.04% and 19.47%) (Table 3). Peng et al.^32^ reported the following CVs from fatty acids in *M. oleifera* seed oil: octadecenoic acid (7.32%), docosenoic acid (7.44%), nervonic acid (8.30%), docosanoic acid (12.07%), tetracosanoic acid (13.14%), hexadecanoic acid (20.66%), dodecenoic acid (22.95%), arachidic acid (32.68%), octadecadienoic acid (43.34%), and octadecanoic acid (82.79%). Taken together with our results, this suggests that the content of some fatty acids, including nervonic acid, octadecenoic acid, docosenoic acid, is relatively stable in *M. oleifera* seed oil.

The lipid biosynthesis is influenced by environmental factors such as temperature and rainfall^33,34^. Many studies indicated that temperature was negatively correlated with seed oil concentration^35–37^. Qiao et al.^38^ reported that seed oil content of *Acer truncatum* showed significant negative correlations with both annual average temperature and annual rainfall. Nevertheless, some studies showed that seed oil content increased with the optimum temperature, above which the oil concentration declined^39–41^. We found that seed oil content was significantly higher in the samples from Funing than those from Guangnan (Table 2). This showed higher temperature could improve seed oil content of *M. oleifera*. Composition of fatty acid varied with temperature and rainfall. Temperature plays a leading role in modifying oilseed rape quantity and the balance between saturated, mono-unsaturated and polyunsaturated fatty acids^42^. The saturated fatty acids such as octadecanoic acid, arachidic acid, docosanoic acid, and tetracosanoic acid, were higher in warm climates, while mono-unsaturated fatty acids did not demonstrate a uniform response to temperature in *Camelina sativa*, as demonstrated by increasing octadecenoic acid and docosenoic acid with temperature, and decreasing hexadecenoic acid and dodecenoic acid with temperature^33^. Octadecenoic acid was negatively correlated, while hexadecanoic acid and Octadecadienoic acid were positively correlated with the mean daily temperature at the time of intense growth and ripening of the olive fruits^34^. Other reports showed that nervonic acid content was not influenced by temperature in *Acer truncatum* and *Camelina sativa*^33,38,43^. However, precipitation exhibited a significant positive correlation with nervonic acid in *Acer truncatum*^38^. It was also found that weather conditions deviating from the long-term average, both in terms of temperature and precipitation, did not affect the quantitative profile of fatty acids^44^. Our studies showed nervonic acid content was significantly higher in *M. oleifera* in Funing, while the other main fatty acids (content > 1%) was significantly lower except for octadecenoic acid and tetracosanoic acid (Table 3). The results suggest that both seed oil production and quality may be improved by planting *M. oleifera* in warmer areas.

### Correlations between fruit traits

**Figure 2:**
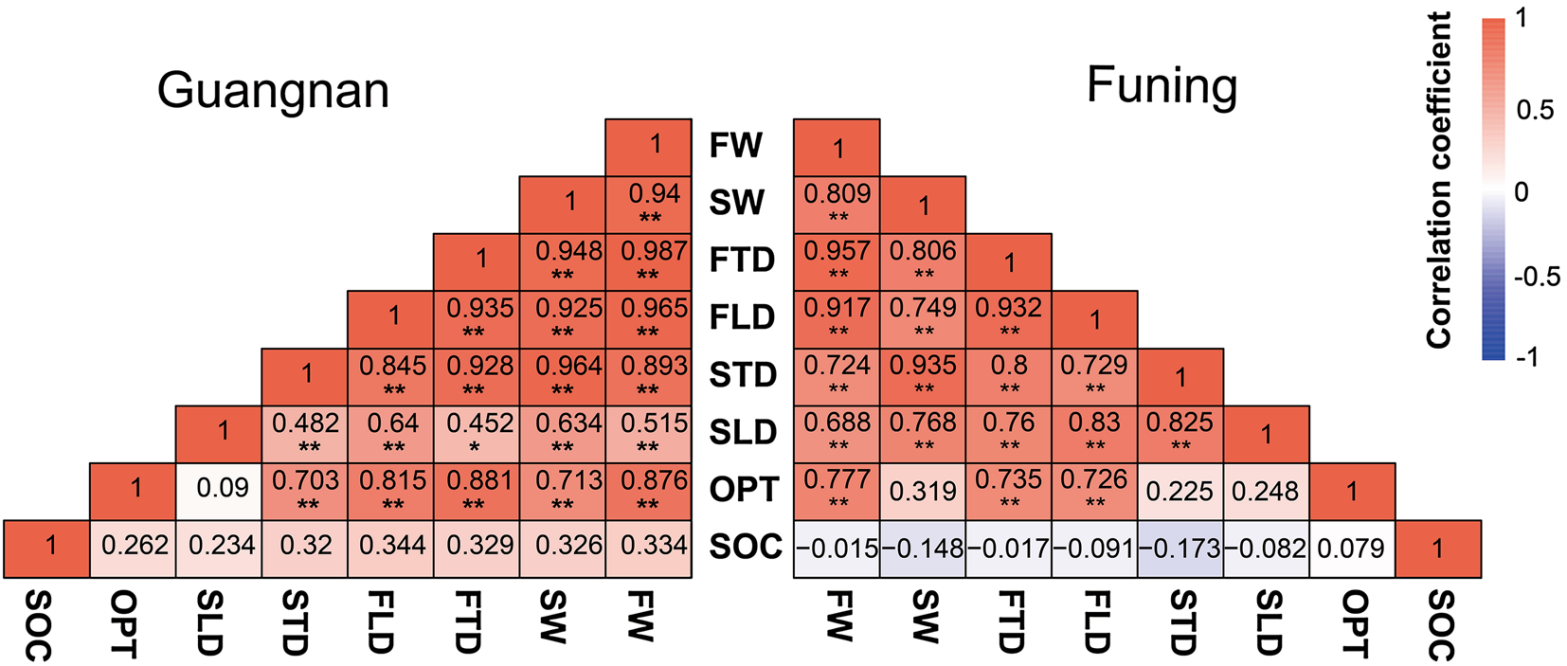
Correlations between fruit traits of individuals in Guangnan and Funing. *FW* fruit weight, *SW* stone weight, *FTD* mean fruit transverse diameter, *FLD* mean fruit longitudinal diameter, *STD* mean stone transverse diameter, *SLD* mean stone longitudinal diameter, *OPT* the outer pericarp thickness, *SOC* seed oil content. *p < 0.05; **p < 0.01.

There were significant positive correlations between every two traits (fruit weight, stone weight, fruit transverse diameter, fruit longitudinal diameter, stone transverse diameter, and stone longitudinal diameter) both at Guangnan or Funing (Fig. 2). The thickness of the outer pericarp was positively and significantly correlated with both the weight and dimensions of fruits at both of the sites, however, the thickness of the outer pericarp was only positively and significantly correlated with the weight and transverse diameter of the stone at Guangnan, having no correlation with stone characters at Funing (Fig. 2). Lv et al.^20^ reported that the proportion of the outer pericarp weight increased with increased fruit weight. This conclusion corresponds with our findings that the thickness of the outer pericarp correlates more with the weight and dimensions of the fruit than with those of the stone. Seed oil content did not correlate with the weights or dimensions of either the fruit or the stone. This demonstrates that the morphological characters of the fruit and stone do not have a great effect on the seed oil content. There was usually an extremely significant correlation between the phenotypic fruiting traits in drupe plants, such as fruit weight and dimension, stone weight and dimension, and pulp thickness^26,45^. Therefore, when selecting plants with elite traits, we should mainly focus on some representative indicators. For the selection of high-quality individuals of *M. oleifera*, large fruit, large stone and high oil content are indicators of elite traits.

**Figure 3:**
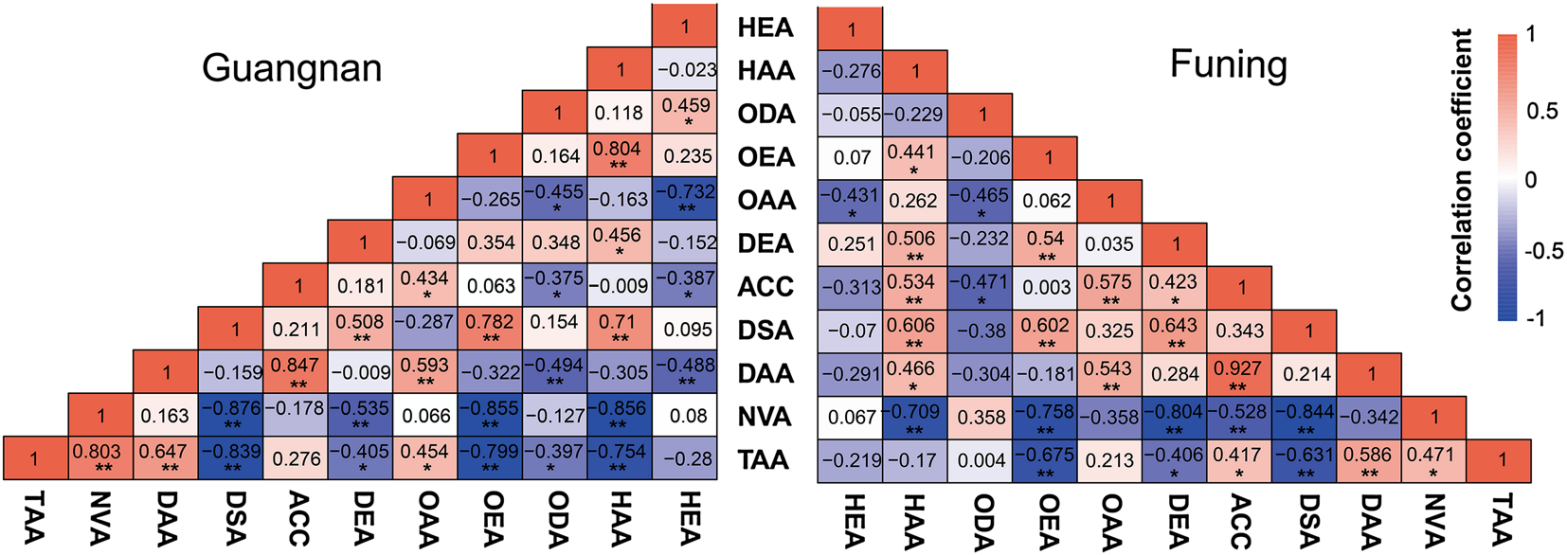
Correlations between fatty acid components in the seed oil from individuals in Guangnan and Funing. *HEA* hexadecenoic acid, *HAA* hexadecanoic acid, *ODA* octadecadienoic acid, *OEA* octadecenoic acid, *OAA* octadecanoic acid, *DEA* dodecenoic acid, *ACC* arachidic acid, *DSA* docosenoic acid, *DAA* docosanoic acid, *NVA* nervonic acid, *TAA* tetracosanoic acid. *p < 0.05; **p < 0.01.

The amounts of nervonic acid and tetracosanoic acid were significantly and negatively correlated with the major fatty acids (octadecenoic acid, dodecenoic acid and docosenoic acid), but there was a significant and positive correlation between the content of nervonic acid and tetracosanoic acid in *M. oleifera* seed oil both at Guangnan and Funing (Fig. 3). Docosenoic acid had a significant and positive correlation with octadecenoic acid and dodecenoic acid at both sites. The correlation between octadecenoic acid and dodecenoic acid was not significant at Guangnan, but it was both significant and positive at Funing (Fig. 3). It was worth noting that the correlation between nervonic acid and the other major fatty acids was very similar in the samples from both Guangnan and Funing. The biosynthetic pathway of nervonic acid includes de novo fatty acid synthesis in plant plastids and fatty acid elongation starting from C18:1, and nervonic acid as a long-chain fatty acids is synthesized in the form of acyl-CoAs by the fatty acid elongation enzyme complex, C20:1 and C22:1 are the precursors in the synthesis of nervonic acid^46^. This may be the reason why nervonic acid is highly and negatively correlated with these major fatty acids.

With rigorous investigation and research, some endemic and endangered plant species are subsequently discovered and re-understood, some of which possess great utilization value in medicine, food, and as ornamental, etc^47,48^. Due to the vulnerability of endemic species in environmental adaptation, it is not easy to propagate and cultivate these species^49^, restricting the development and utilization of these species. Usually, some wild plant resources are collected in large quantities for direct use or economic purposes, which could accelerate the endangerment and extinction of these species^50,51^. By selecting good genotype suitable for domestication and using appropriate cultivation techniques, there are good prospects in using these plants and serving the local economy, and preserving wild resources.

## Conclusion

*Malania oleifera* is considered to be a potentially important resource for the development of nervonic acid products. The variability of characters related to fruiting is able to demonstrate the diversity in this wild resource. Significant differences in fruiting characters, including SW, FTD, FLD, SLD, or OPT, were found among individual trees, and these differences were greater than between the populations of this species at two sites with different climates. Fruit weight, stone weight and outer pericarp thickness had the highest CV values. The seed oil content from trees growing in Funing (which had a higher mean temperature than Guangnan) was significantly higher (*p* < 0.01) than that from trees growing in Guangnan. Eleven fatty acids were identified from the seed oil, and the three present in the largest amounts were nervonic acid, octadecenoic acid and docosenoic acid, together representing 73–92% of the total seed oil in individuals. Nervonic acid was present in significantly higher amount (*p* < 0.01) in seed oil from trees in Funing. Although the correlations between the weight and dimensions of fruits and stones were significantly different (*p* < 0.01 or *p* < 0.05), these characters did not correlate in any way with seed oil content (*p* > 0.05). The differences in fruit morphological characters were weak between Guangnan and Funing, but seed oil and nervonic content were significantly higher from trees in Funing than those in Guangnan, suggesting that cultivation of *M. oleifera* may yield a better economic harvest in areas with higher mean annual climatic temperatures. We hope that this study can assist the selection of appropriate *M. oleifera* individuals to breed robust varieties for cultivation.

## Supplementary Information

Supplementary Information.
