# Supplementary material for: Fruiting character variability in wild individuals of Malania oleifera, a highly valued endemic species
Source: Sci Rep. 2021 Dec 8;11:23605. doi: 10.1038/s41598-021-03080-7 (PMC8655003; doi:10.1038/s41598-021-03080-7)
Supplement: Supplementary file 1 — Supplementary Information. [file 41598_2021_3080_MOESM1_ESM.doc]

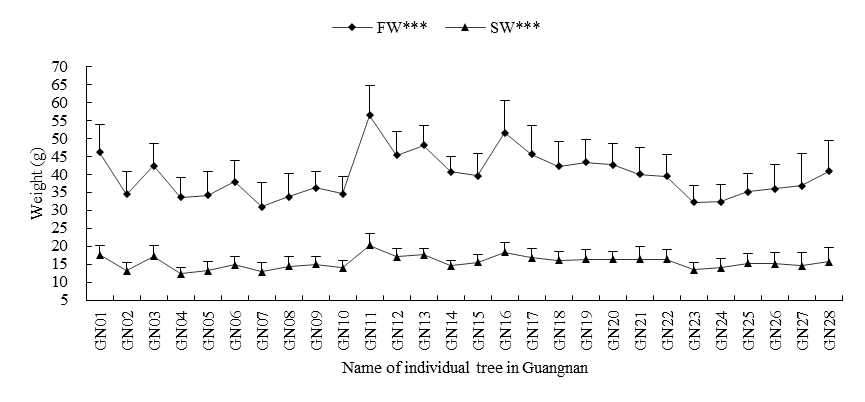


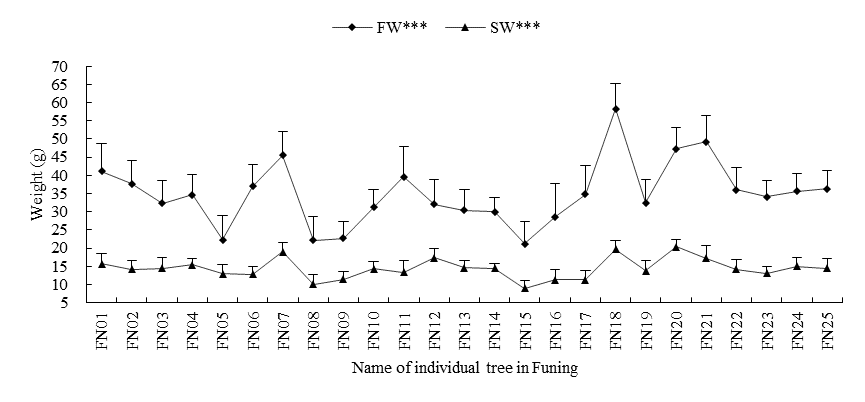


**Figure S1.** Mean fresh fruit and stone weight for individual trees (mean ± SD). FW, mean fruit weight; SW, mean stone weight; *** indicates significant level among individuals (*p*<0.001).


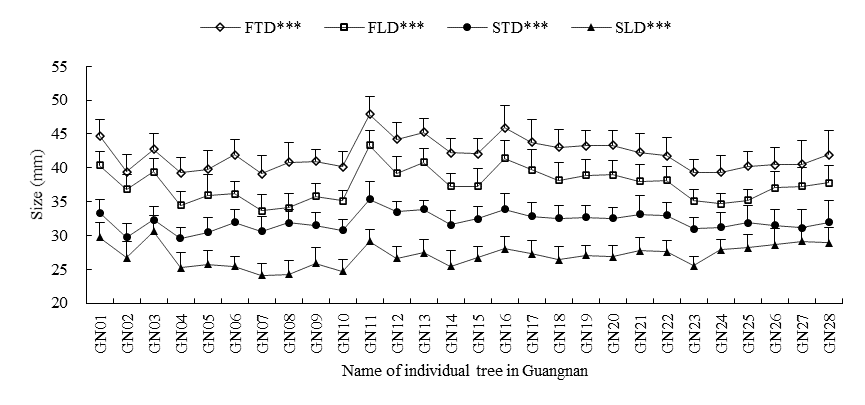


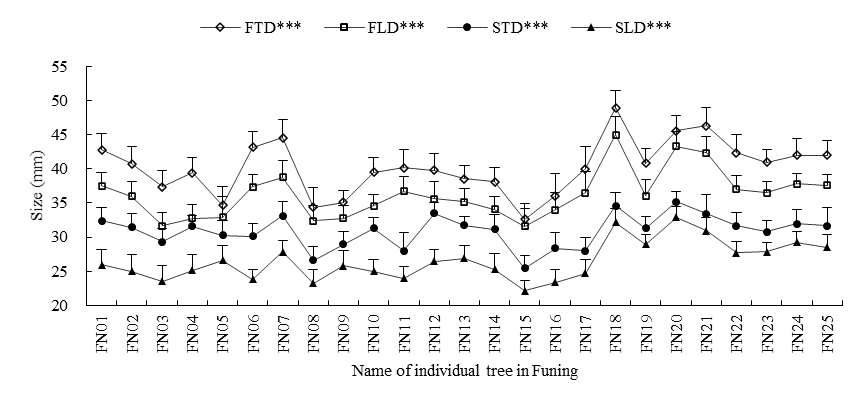


**Figure S2.** Mean fresh fruit and stone dimensions for individual trees. FTD, mean fruit transverse diameter; FLD, mean fruit longitudinal diameter; STD, mean stone transverse diameter; SLD, mean stone longitudinal diameter; *** indicates significant level among individuals (*p*<0.001).


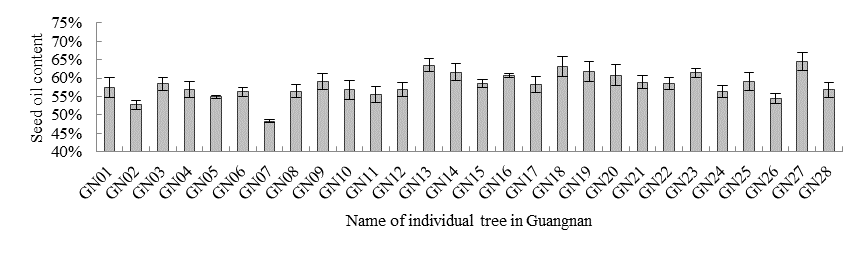

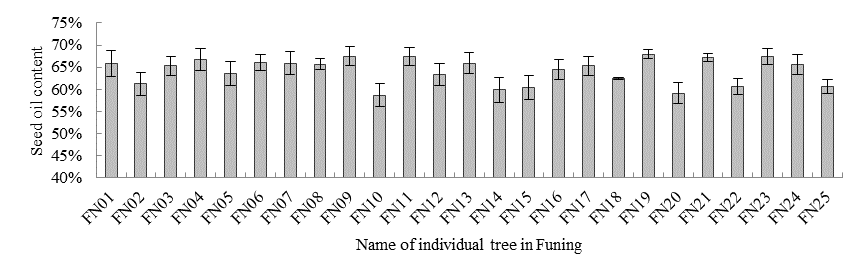


**Figure S3.** Seed oil content for each individual tree

**Table S1.** Comparison of significant differences in the mean weights of the fruit (below the diagonal) and stone (above the diagonal) from individuals of *Malania oleifera* at Guangnan (LSD test)

| Tree | GN01 | GN02 | GN03 | GN04 | GN05 | GN06 | GN07 | GN08 | GN09 | GN10 | GN11 | GN12 | GN13 | GN14 |
| --- | --- | --- | --- | --- | --- | --- | --- | --- | --- | --- | --- | --- | --- | --- |
| GN01 | ---- | 4.38^***^ | 0.37^NS^ | 5.29^***^ | 4.35^***^ | 2.73^***^ | 4.74^***^ | 3.15^***^ | 2.64^***^ | 3.61^***^ | 2.65^***^ | 0.51^NS^ | 0.08^NS^ | 3.01^***^ |
| GN02 | 11.61^***^ | ---- | 4.01^***^ | 0.90^NS^ | 0.03^NS^ | 1.65^**^ | 0.35^NS^ | 1.23^*^ | 1.74^***^ | 0.78^NS^ | 7.03^***^ | 3.87^***^ | 4.46^***^ | 1.38^**^ |
| GN03 | 3.69^**^ | 7.91^***^ | ---- | 4.92^***^ | 3.98^***^ | 2.36^***^ | 4.37^***^ | 2.78^***^ | 2.28^***^ | 3.24^***^ | 3.02^***^ | 0.14^NS^ | 0.45^NS^ | 2.64^***^ |
| GN04 | 12.51^***^ | 0.90^NS^ | 8.81^***^ | ---- | 0.93^NS^ | 2.55^***^ | 0.55^NS^ | 2.14^***^ | 2.64^***^ | 1.68^**^ | 7.94^***^ | 4.78^***^ | 5.36^***^ | 2.28^***^ |
| GN05 | 11.94^***^ | 0.33^NS^ | 8.24^***^ | 0.57^NS^ | ---- | 1.62^**^ | 0.38^NS^ | 1.20^*^ | 1.71^**^ | 0.75^**^ | 7.00^***^ | 3.84^***^ | 4.43^***^ | 1.35^*^ |
| GN06 | 8.10^***^ | 3.51^**^ | 4.40^***^ | 4.41^***^ | 3.83^**^ | ---- | 2.00^***^ | 0.42^NS^ | 0.09^NS^ | 0.87^NS^ | 5.38^***^ | 2.22^***^ | 2.81^***^ | 0.27^NS^ |
| GN07 | 15.05^***^ | 3.44^**^ | 11.35^***^ | 2.54^*^ | 3.11^*^ | 6.94^***^ | ---- | 1.59^**^ | 2.09^***^ | 1.13^*^ | 7.39^***^ | 4.23^***^ | 4.81^***^ | 1.73^***^ |
| GN08 | 12.34^***^ | 0.73^NS^ | 8.64^***^ | 0.17^NS^ | 0.40^NS^ | 4.24^**^ | 2.71^*^ | ---- | 0.50^NS^ | 0.45^NS^ | 5.80^***^ | 2.64^***^ | 3.23^***^ | 0.15^NS^ |
| GN09 | 9.87^***^ | 1.74^NS^ | 6.18^***^ | 2.64^*^ | 2.06^NS^ | 1.77^NS^ | 5.17^***^ | 2.46^**^ | ---- | 0.96^NS^ | 5.30^***^ | 2.14^***^ | 2.72^***^ | 0.36^NS^ |
| GN10 | 11.49^***^ | 0.12^NS^ | 7.80^***^ | 1.02^NS^ | 0.44^NS^ | 3.39^**^ | 3.55^**^ | 0.84^NS^ | 1.62^NS^ | ---- | 6.25^***^ | 3.10^***^ | 3.68^***^ | 0.60^NS^ |
| GN11 | 10.35^***^ | 21.96^***^ | 14.05^***^ | 22.86^***^ | 22.29^***^ | 18.45^***^ | 25.40^***^ | 22.69^***^ | 20.22^***^ | 21.85^***^ | ---- | 3.16^***^ | 2.57^***^ | 5.65^***^ |
| GN12 | 0.77^NS^ | 10.83^***^ | 2.92^*^ | 11.74^***^ | 11.16^***^ | 7.32^***^ | 14.27^***^ | 11.56^***^ | 9.10^***^ | 10.72^***^ | 11.13^***^ | ---- | 0.59^NS^ | 2.50^***^ |
| GN13 | 1.94^NS^ | 13.54^***^ | 5.63^***^ | 14.45^***^ | 13.87^***^ | 10.04^***^ | 16.98^***^ | 14.27^***^ | 11.81^***^ | 13.43^***^ | 8.42^***^ | 2.71^*^ | ---- | 3.08^***^ |
| GN14 | 5.31^***^ | 6.30^***^ | 1.61^NS^ | 7.20^***^ | 6.63^***^ | 2.79^*^ | 9.74^***^ | 7.03^***^ | 4.57^**^ | 6.19^***^ | 15.66^***^ | 4.53^***^ | 7.24^***^ | ---- |
| GN15 | 6.45^***^ | 5.16^***^ | 2.75^*^ | 6.06^***^ | 5.49^***^ | 1.65^NS^ | 8.60^***^ | 5.89^***^ | 3.42^**^ | 5.04^***^ | 16.80^***^ | 5.67^***^ | 8.39^***^ | 1.142^NS^ |
| GN16 | 5.53^***^ | 17.13^***^ | 9.22^***^ | 18.04^***^ | 17.46^***^ | 13.63^***^ | 20.57^***^ | 17.86^***^ | 15.40^***^ | 17.01^***^ | 4.83^***^ | 6.30^***^ | 3.59^**^ | 10.83^***^ |
| GN17 | 0.47^NS^ | 11.14^***^ | 3.21^*^ | 12.04^***^ | 11.47^***^ | 7.63^***^ | 14.58^***^ | 11.87^***^ | 9.40^***^ | 11.02^***^ | 10.82^***^ | 0.31^NS^ | 2.41^NS^ | 4.84^***^ |
| GN18 | 3.83^**^ | 7.78^***^ | 0.13^NS^ | 8.68^***^ | 8.11^***^ | 4.27^***^ | 11.22^***^ | 8.51^***^ | 6.04^***^ | 7.66^***^ | 14.18^***^ | 3.05^*^ | 5.77^***^ | 1.48^NS^ |
| GN19 | 2.72^*^ | 8.89^***^ | 0.98^NS^ | 9.79^***^ | 9.22^***^ | 5.38^***^ | 12.33^***^ | 9.62^**^ | 7.15^***^ | 8.77^***^ | 13.07^***^ | 1.94^NS^ | 4.66^***^ | 2.59^*^ |
| GN20 | 3.36^**^ | 8.24^***^ | 0.33^NS^ | 9.14^***^ | 8.57^***^ | 4.73^***^ | 11.68^***^ | 8.97^**^ | 6.51^**^ | 8.13^***^ | 13.71^***^ | 2.59^*^ | 5.30^***^ | 1.94^NS^ |
| GN21 | 6.03^***^ | 5.58^***^ | 2.33^NS^ | 6.48^***^ | 5.91^***^ | 2.07^NS^ | 9.02^***^ | 6.31^***^ | 3.85^**^ | 5.47^***^ | 16.37^***^ | 5.25^***^ | 7.96^***^ | 0.72^NS^ |
| GN22 | 6.54^***^ | 5.06^***^ | 2.85^*^ | 5.96^***^ | 5.39^***^ | 1.55^NS^ | 8.50^***^ | 5.79^***^ | 3.31^*^ | 4.95^***^ | 16.90^***^ | 5.77^***^ | 8.48^***^ | 1.24^NS^ |
| GN23 | 13.79^***^ | 2.18^NS^ | 10.09^***^ | 1.28^NS^ | 1.85^NS^ | 5.69^***^ | 1.26^NS^ | 1.45^NS^ | 3.91^**^ | 2.30^NS^ | 24.14^***^ | 13.02^***^ | 15.73^***^ | 8.48^***^ |
| GN24 | 13.71^***^ | 2.10^NS^ | 10.01^***^ | 1.19^NS^ | 1.77^NS^ | 5.61^***^ | 1.34^NS^ | 1.37^NS^ | 3.83^**^ | 2.21^NS^ | 24.06^***^ | 12.93^***^ | 15.65^***^ | 8.40^***^ |
| GN25 | 10.86^***^ | 0.74^NS^ | 7.17^***^ | 1.64^NS^ | 1.07^NS^ | 2.76^*^ | 4.18^**^ | 1.47^NS^ | 0.99^NS^ | 0.63^NS^ | 21.22^***^ | 10.09^***^ | 12.80^***^ | 5.56^***^ |
| GN26 | 10.12^***^ | 1.49^NS^ | 6.42^***^ | 2.39^NS^ | 1.82^NS^ | 2.02^NS^ | 4.93^***^ | 2.22^NS^ | 0.24^NS^ | 1.38^NS^ | 20.47^***^ | 9.34^***^ | 12.05^***^ | 4.81^***^ |
| GN27 | 9.24^***^ | 2.37^NS^ | 5.54^***^ | 3.27^*^ | 2.70^*^ | 1.14^NS^ | 5.81^***^ | 3.10^*^ | 0.63^NS^ | 2.25^NS^ | 19.59^***^ | 8.46^***^ | 11.18^***^ | 3.93^**^ |
| GN28 | 5.16^***^ | 6.45^***^ | 1.46^NS^ | 7.35^***^ | 6.78^***^ | 2.94^*^ | 9.89^***^ | 7.18^***^ | 4.71^***^ | 6.33^***^ | 15.51^***^ | 4.38^***^ | 7.10^***^ | 0.15^NS^ |

NS *p* > 0.05, * *p* ≤ 0.05, ** *p* ≤ 0.01, *** *p* ≤ 0.001

**Table S1.** (continued)

| Tree | GN15 | GN16 | GN17 | GN18 | GN19 | GN20 | GN21 | GN22 | GN23 | GN24 | GN25 | GN26 | GN27 | GN28 |
| --- | --- | --- | --- | --- | --- | --- | --- | --- | --- | --- | --- | --- | --- | --- |
| GN01 | 2.01^***^ | 0.70^NS^ | 0.84^NS^ | 1.44^**^ | 1.25^*^ | 1.19^*^ | 1.14^*^ | 1.21^*^ | 4.16^***^ | 3.53^***^ | 2.35^***^ | 2.52^***^ | 2.95^***^ | 1.88^***^ |
| GN02 | 2.37^***^ | 5.08^***^ | 3.55^***^ | 2.94^***^ | 3.13^***^ | 3.19^***^ | 3.25^***^ | 3.18^**^ | 0.22^NS^ | 0.85^NS^ | 2.04^***^ | 1.87^***^ | 1.43^**^ | 2.50^***^ |
| GN03 | 1.64^**^ | 1.07^*^ | 0.47^NS^ | 1.07^*^ | 0.89^NS^ | 0.82^NS^ | 0.77^NS^ | 0.84^NS^ | 3.79^***^ | 3.16^***^ | 1.99^***^ | 2.15^***^ | 2.58^***^ | 1.52^**^ |
| GN04 | 3.28^***^ | 5.98^***^ | 4.45^***^ | 3.85^***^ | 4.03^***^ | 4.09^***^ | 4.15^***^ | 4.08^***^ | 1.12^*^ | 1.76^***^ | 2.94^***^ | 2.77^***^ | 2.34^***^ | 3.40^***^ |
| GN05 | 2.34^***^ | 5.05^***^ | 3.52^***^ | 2.91^***^ | 3.10^***^ | 3.16^***^ | 3.22^***^ | 3.15^***^ | 0.19^NS^ | 0.82^NS^ | 2.01^***^ | 1.84^***^ | 1.40^**^ | 2.47^***^ |
| GN06 | 0.72^NS^ | 3.43^***^ | 1.90^***^ | 1.29^*^ | 1.48^**^ | 1.54^**^ | 1.60^**^ | 1.52^**^ | 1.43^**^ | 0.80^NS^ | 0.39^NS^ | 0.22^NS^ | 0.22^NS^ | 0.85^NS^ |
| GN07 | 2.73^***^ | 5.44^***^ | 3.90^***^ | 3.30^****^ | 3.48^**^ | 3.54^***^ | 3.60^***^ | 3.53^***^ | 0.57^NS^ | 1.21^*^ | 2.39^***^ | 2.22^***^ | 1.79^***^ | 2.85^***^ |
| GN08 | 1.14^*^ | 3.85^***^ | 2.32^***^ | 1.71^***^ | 1.90^***^ | 1.96^***^ | 2.01^***^ | 1.94^***^ | 1.01^NS^ | 0.38^NS^ | 0.80^NS^ | 0.63^NS^ | 0.20^NS^ | 1.27^*^ |
| GN09 | 0.64^NS^ | 3.35^***^ | 1.81^***^ | 1.21^**^ | 1.39^**^ | 1.45^**^ | 1.51^**^ | 1.44^**^ | 1.52^**^ | 0.88^NS^ | 0.30^NS^ | 0.13^NS^ | 0.30^NS^ | 0.76^NS^ |
| GN10 | 1.59^**^ | 4.30^***^ | 2.77^***^ | 2.17^***^ | 2.35^***^ | 2.41^***^ | 2.47^***^ | 2.40^***^ | 0.56^NS^ | 0.08^NS^ | 1.26^*^ | 1.09^*^ | 0.65^NS^ | 1.72^***^ |
| GN11 | 4.66^***^ | 1.95^***^ | 3.48^***^ | 4.09^***^ | 3.90^***^ | 3.84^***^ | 3.79^***^ | 3.86^***^ | 6.81^***^ | 6.18^***^ | 5.00^***^ | 5.17^***^ | 5.60^***^ | 4.53^***^ |
| GN12 | 1.50^**^ | 1.21^*^ | 0.33^NS^ | 0.93^NS^ | 0.74^NS^ | 0.68^NS^ | 0.63^NS^ | 0.70^NS^ | 3.65^***^ | 3.02^***^ | 1.84^***^ | 2.01^***^ | 2.44^***^ | 1.37^**^ |
| GN13 | 2.09^***^ | 0.62^NS^ | 0.91^NS^ | 1.52^**^ | 1.33^*^ | 1.27^*^ | 1.21^*^ | 1.29^*^ | 4.24^***^ | 3.61^***^ | 3.42^***^ | 2.59^***^ | 3.03^***^ | 1.96^***^ |
| GN14 | 1.00^NS^ | 3.70^***^ | 2.17^***^ | 1.57^**^ | 1.75^***^ | 1.81^***^ | 1.87^***^ | 1.80^***^ | 1.16^*^ | 0.52^NS^ | 0.66^NS^ | 0.49^NS^ | 0.06^NS^ | 1.12^*^ |
| GN15 | ---- | 2.71^***^ | 1.17^*^ | 0.57^NS^ | 0.76^NS^ | 0.82^NS^ | 0.87^NS^ | 0.80^NS^ | 2.15^***^ | 1.51^**^ | 0.34^NS^ | 0.51^NS^ | 0.94^NS^ | 0.13^NS^ |
| GN16 | 11.97^***^ | ---- | 1.53^**^ | 2.14^***^ | 1.95^***^ | 1.89^***^ | 1.83^***^ | 1.91^***^ | 4.68^***^ | 4.22^***^ | 3.05^***^ | 3.22^***^ | 3.65^***^ | 2.58^***^ |
| GN17 | 5.98^***^ | 6.00^***^ | ---- | 0.60^NS^ | 0.42^NS^ | 0.36^NS^ | 0.30^NS^ | 0.37^NS^ | 3.33^***^ | 2.69^***^ | 1.51^**^ | 1.68^**^ | 2.11^***^ | 1.05^*^ |
| GN18 | 2.62^*^ | 9.36^***^ | 3.36^*^ | ---- | 0.19^NS^ | 0.25^NS^ | 0.30^NS^ | 0.23^NS^ | 2.72^***^ | 2.09^***^ | 0.91^NS^ | 1.08^*^ | 1.51^**^ | 0.45^NS^ |
| GN19 | 3.73^**^ | 8.25^***^ | 2.25^NS^ | 1.11^NS^ | ---- | 0.06^NS^ | 0.12^NS^ | 0.05^NS^ | 2.91^***^ | 2.28^***^ | 1.09^*^ | 1.26^*^ | 1.70^***^ | 0.63^NS^ |
| GN20 | 3.08^*^ | 8.89^***^ | 2.90^*^ | 0.46^NS^ | 0.65^NS^ | ---- | 0.06^NS^ | 0.02^NS^ | 2.97^***^ | 2.34^***^ | 1.15^*^ | 1.32^*^ | 1.76^***^ | 0.69^NS^ |
| GN21 | 0.42^NS^ | 11.55^***^ | 5.56^***^ | 2.20^NS^ | 3.31^*^ | 2.66^*^ | ---- | 0.07^NS^ | 3.03^***^ | 2.39^***^ | 1.21^*^ | 1.38^**^ | 1.81^***^ | 0.75^NS^ |
| GN22 | 0.10^NS^ | 12.07^***^ | 6.08^***^ | 2.72^*^ | 3.83^**^ | 3.18^*^ | 0.52^NS^ | ---- | 2.96^***^ | 2.32^***^ | 1.14^*^ | 1.31^*^ | 1.74^***^ | 0.68^NS^ |
| GN23 | 7.34^***^ | 19.32^***^ | 13.32^***^ | 9.96^***^ | 11.07^***^ | 10.42^***^ | 7.76^***^ | 7.24^***^ | ---- | 0.63^NS^ | 1.81^**^ | 1.65^***^ | 1.21^*^ | 2.28^***^ |
| GN24 | 7.26^***^ | 19.24^***^ | 13.24^***^ | 9.88^***^ | 10.99^***^ | 10.34^***^ | 7.68^***^ | 7.16^***^ | 0.08^NS^ | ---- | 1.18^*^ | 1.01^NS^ | 0.58^NS^ | 1.64^**^ |
| GN25 | 4.41^***^ | 16.39^***^ | 10.39^***^ | 7.03^***^ | 8.14^***^ | 7.50^***^ | 4.84^***^ | 4.32^***^ | 2.93^*^ | 2.85^*^ | ---- | 0.17^NS^ | 0.60^NS^ | 0.46^NS^ |
| GN26 | 3.67^**^ | 15.64^***^ | 9.65^***^ | 6.29^***^ | 7.40^***^ | 6.75^***^ | 4.09^**^ | 3.57^**^ | 3.67^**^ | 3.59^**^ | 0.75^NS^ | ---- | 0.43^NS^ | 0.63^NS^ |
| GN27 | 2.79^*^ | 14.77^***^ | 8.77^***^ | 5.41^***^ | 6.52^***^ | 5.87^***^ | 3.21^**^ | 2.69^*^ | 4.55^***^ | 4.47^***^ | 1.62^NS^ | 0.88^NS^ | ---- | 1.07^*^ |
| GN28 | 1.29^NS^ | 10.68^***^ | 4.69^***^ | 1.33^NS*^ | 2.44^NS^ | 1.79^NS^ | 0.87^NS^ | 1.39^NS^ | 8.63^***^ | 8.55^***^ | 5.70^***^ | 4.96^***^ | 4.08^**^ | ---- |

**Table S2.** Comparison of significant differences in mean fruit transverse diameter (below the diagonal) and longitudinal diameter (above the diagonal) of individuals of *Malania oleifera* at Guangnan (LSD test)

| Tree | GN01 | GN02 | GN03 | GN04 | GN05 | GN06 | GN07 | GN08 | GN09 | GN10 | GN11 | GN12 | GN13 | GN14 |
| --- | --- | --- | --- | --- | --- | --- | --- | --- | --- | --- | --- | --- | --- | --- |
| GN01 | ---- | 3.58^**^ | 0.96^NS^ | 5.93^***^ | 4.47^***^ | 4.21^***^ | 6.78^***^ | 6.31^***^ | 4.59^***^ | 5.28^***^ | 2.91^***^ | 1.24^**^ | 0.42^NS^ | 3.11^***^ |
| GN02 | 5.33^***^ | ---- | 2.62^***^ | 2.35^***^ | 0.90^*^ | 0.63^NS^ | 3.20^***^ | 2.73^***^ | 1.01^*^ | 1.70^***^ | 6.48^***^ | 2.34^***^ | 3.99^***^ | 0.46^NS^ |
| GN03 | 1.97^***^ | 3.36^***^ | ---- | 4.97^***^ | 3.51^***^ | 3.25^***^ | 5.82^***^ | 5.35^***^ | 3.63^***^ | 4.32^***^ | 3.87^***^ | 0.28^NS^ | 1.38^**^ | 2.15^**^ |
| GN04 | 5.46^***^ | 0.13^NS^ | 3.49^***^ | ---- | 1.46^***^ | 1.72^***^ | 0.85^NS^ | 0.38^NS^ | 1.34^**^ | 0.65^NS^ | 8.84^***^ | 4.69^***^ | 6.35^***^ | 2.81^***^ |
| GN05 | 4.91^***^ | 0.42^NS^ | 2.94^***^ | 0.55^NS^ | ---- | 0.26^NS^ | 2.30^***^ | 1.83^***^ | 0.12^NS^ | 0.81^NS^ | 7.38^***^ | 3.24^***^ | 4.89^***^ | 1.36^**^ |
| GN06 | 2.82^***^ | 2.52^***^ | 0.85^NS^ | 2.65^***^ | 2.09^***^ | ---- | 2.57^***^ | 2.10^***^ | 0.38^NS^ | 1.07^*^ | 7.12^***^ | 2.97^***^ | 4.63^***^ | 1.10^*^ |
| GN07 | 5.57^***^ | 0.23^NS^ | 3.60^***^ | 0.10^NS^ | 0.66^NS^ | 2.75^***^ | ---- | 0.47^NS^ | 2.18^***^ | 1.50^***^ | 9.68^***^ | 5.54^***^ | 7.19^***^ | 3.66^***^ |
| GN08 | 3.88^***^ | 1.46^**^ | 1.91^***^ | 1.59^**^ | 1.03^*^ | 1.06^*^ | 1.69^***^ | ---- | 1.72^***^ | 1.03^*^ | 9.22^***^ | 5.07^***^ | 6.73^***^ | 3.19^***^ |
| GN09 | 3.73^***^ | 1.61^**^ | 1.76^***^ | 1.74^***^ | 1.18^*^ | 0.91^NS^ | 1.84^***^ | 0.15^NS^ | ---- | 0.69^NS^ | 7.50^***^ | 3.35^***^ | 5.01^***^ | 1.47^***^ |
| GN10 | 4.56^***^ | 0.77^NS^ | 2.59^***^ | 0.90^NS^ | 0.35^NS^ | 1.75^**^ | 1.01^*^ | 0.69^NS^ | 0.84^NS^ | ---- | 8.19^***^ | 4.04^***^ | 5.70^***^ | 2.17^***^ |
| GN11 | 3.18^***^ | 8.51^***^ | 5.15^***^ | 8.64^***^ | 8.09^***^ | 6.00^***^ | 8.75^***^ | 7.06^***^ | 6.91^***^ | 7.74^***^ | ---- | 4.15^***^ | 2.49^***^ | 6.02^***^ |
| GN12 | 0.47^NS^ | 4.86^***^ | 1.50^**^ | 5.00^***^ | 4.44^***^ | 2.35^***^ | 5.10^***^ | 3.41^***^ | 3.26^***^ | 4.09^***^ | 3.65^***^ | ---- | 1.66^***^ | 1.88^***^ |
| GN13 | 0.54^NS^ | 5.88^***^ | 2.51^***^ | 6.01^***^ | 5.45^***^ | 3.36^***^ | 6.11^***^ | 4.42^***^ | 4.27^***^ | 5.11^***^ | 2.64^***^ | 1.01^*^ | ---- | 3.53^***^ |
| GN14 | 2.47^***^ | 2.86^***^ | 0.50^NS^ | 2.99^***^ | 2.44^***^ | 0.35^NS^ | 3.10^***^ | 1.41^**^ | 1.26^*^ | 2.09^***^ | 5.65^***^ | 2.00^***^ | 3.01^***^ | ---- |
| GN15 | 2.65^***^ | 2.68^***^ | 0.68^NS^ | 2.81^***^ | 2.26^***^ | 0.17^NS^ | 2.92^***^ | 1.23^*^ | 1.08^*^ | 1.91^***^ | 5.83^***^ | 2.18^***^ | 3.19^***^ | 0.18^NS^ |
| GN16 | 1.23^*^ | 6.56^***^ | 3.20^***^ | 6.69^***^ | 6.14^***^ | 4.05^***^ | 6.80^***^ | 5.11^***^ | 4.96^***^ | 5.79^***^ | 1.95^***^ | 1.70^***^ | 0.69^NS^ | 3.70^***^ |
| GN17 | 0.95^NS^ | 4.38^***^ | 1.03^*^ | 4.52^***^ | 3.96^***^ | 1.87^***^ | 4.62^***^ | 2.93^***^ | 2.78^***^ | 3.62^***^ | 4.13^***^ | 0.48^NS^ | 1.49^**^ | 1.53^**^ |
| GN18 | 1.66^***^ | 3.68^***^ | 0.32^NS^ | 3.81^***^ | 3.25^***^ | 1.16^*^ | 3.91^***^ | 2.22^***^ | 2.07^***^ | 2.91^***^ | 4.84^***^ | 1.19^*^ | 2.20^***^ | 0.82^**^ |
| GN19 | 1.43^**^ | 3.91^***^ | 0.55^NS^ | 4.04^***^ | 3.48^***^ | 1.39^**^ | 4.14^***^ | 2.45^***^ | 2.30^***^ | 3.14^***^ | 4.61^***^ | 0.96^NS^ | 1.97^***^ | 1.05^*^ |
| GN20 | 1.39^**^ | 3.94^***^ | 0.58^NS^ | 4.07^***^ | 3.52^***^ | 1.42^**^ | 4.18^***^ | 2.48^***^ | 2.34^***^ | 3.17^***^ | 4.57^***^ | 0.92^NS^ | 1.94^***^ | 1.08^*^ |
| GN21 | 2.43^***^ | 2.90^***^ | 0.46^NS^ | 3.03^***^ | 2.48^***^ | 0.39^NS^ | 3.14^***^ | 1.45^**^ | 1.30^*^ | 2.13^***^ | 5.61^***^ | 1.96^***^ | 2.97^***^ | 0.04^NS^ |
| GN22 | 2.96^***^ | 2.37^***^ | 0.99^NS^ | 2.50^***^ | 1.95^***^ | 0.14^NS^ | 2.61^***^ | 0.92^NS^ | 0.77^NS^ | 1.60^**^ | 6.14^***^ | 2.49^***^ | 3.50^***^ | 0.49^NS^ |
| GN23 | 5.38^***^ | 0.05^NS^ | 3.41^***^ | 0.08^NS^ | 0.47^NS*^ | 2.57^***^ | 0.19^NS^ | 1.51^**^ | 1.65^***^ | 0.82^NS^ | 8.56^***^ | 4.91^***^ | 5.92^***^ | 2.91^***^ |
| GN24 | 5.41^***^ | 0.07^NS^ | 3.44^***^ | 0.06^NS^ | 0.50^NS^ | 2.59^***^ | 0.16^NS^ | 1.53^**^ | 1.68^***^ | 0.84^NS^ | 8.59^***^ | 4.94^***^ | 5.95^***^ | 2.93^***^ |
| GN25 | 4.47^***^ | 0.87^NS^ | 2.49^***^ | 1.00^NS^ | 0.45^NS^ | 1.65^***^ | 1.10^*^ | 0.59^NS^ | 0.74^NS^ | 0.10^NS^ | 7.64^***^ | 4.00^***^ | 5.01^***^ | 1.99^***^ |
| GN26 | 4.29^***^ | 1.04^*^ | 2.32^***^ | 1.17^*^ | 0.62^NS^ | 1.47^**^ | 1.28^*^ | 0.41^NS^ | 0.56^NS^ | 0.27^NS^ | 7.47^***^ | 3.82^***^ | 4.83^***^ | 1.82^***^ |
| GN27 | 4.20^***^ | 1.13^*^ | 2.23^***^ | 1.26^*^ | 0.71^NS^ | 1.39^**^ | 1.36^**^ | 0.33^NS^ | 0.48^NS^ | 0.36^NS^ | 7.38^***^ | 3.74^***^ | 4.75^***^ | 1.73^***^ |
| GN28 | 2.77^***^ | 2.57^***^ | 0.79^NS^ | 2.70^***^ | 2.14^***^ | 0.05^NS^ | 2.80^***^ | 1.11^*^ | 0.96^NS^ | 1.80^***^ | 5.95^***^ | 2.30^**^ | 3.31^***^ | 0.29^**^ |

NS *p* > 0.05, * *p* ≤ 0.05, ** *p* ≤ 0.01, *** *p* ≤ 0.001

**Table S2.** (continued)

| Tree | GN15 | GN16 | GN17 | GN18 | GN19 | GN20 | GN21 | GN22 | GN23 | GN24 | GN25 | GN26 | GN27 | GN28 |
| --- | --- | --- | --- | --- | --- | --- | --- | --- | --- | --- | --- | --- | --- | --- |
| GN01 | 3.13^***^ | 1.02^*^ | 0.69^NS^ | 2.27^***^ | 1.52^***^ | 1.42^**^ | 2.35^***^ | 2.24^***^ | 5.29^***^ | 5.73^***^ | 5.18^***^ | 3.35^***^ | 3.09^***^ | 2.762^***^ |
| GN02 | 0.45^NS^ | 4.59^***^ | 2.88^***^ | 1.31^**^ | 2.05^***^ | 2.15^***^ | 1.22^**^ | 1.34^**^ | 1.71^***^ | 2.15^***^ | 1.61^***^ | 0.23^NS^ | 0.48^NS^ | 0.96^*^ |
| GN03 | 2.17^***^ | 1.98^***^ | 0.27^NS^ | 1.31^**^ | 0.56^NS^ | 0.46^NS^ | 1.39^**^ | 1.28^**^ | 4.33^***^ | 4.77^***^ | 4.22^***^ | 2.39^***^ | 2.13^***^ | 1.66^***^ |
| GN04 | 2.80^***^ | 6.94^***^ | 5.24^***^ | 3.66^***^ | 4.41^***^ | 4.51^***^ | 3.58^***^ | 3.69^***^ | 0.64^NS^ | 0.20^NS^ | 0.75^NS^ | 2.58^***^ | 2.84^***^ | 3.31^***^ |
| GN05 | 1.34^**^ | 5.49^***^ | 3.38^***^ | 2.20^***^ | 2.95^***^ | 3.05^***^ | 2.12^***^ | 2.24^***^ | 0.81^**^ | 1.26^**^ | 0.71^NS^ | 1.12^*^ | 1.38^**^ | 1.85^***^ |
| GN06 | 1.08^*^ | 5.23^***^ | 3.52^***^ | 1.94^***^ | 2.69^***^ | 2.79^***^ | 1.86^***^ | 1.97^***^ | 1.08^*^ | 1.52^***^ | 0.97^*^ | 0.86^NS^ | 1.12^*^ | 1.59^***^ |
| GN07 | 3.65^***^ | 7.79^***^ | 6.08^***^ | 4.51^***^ | 5.25^***^ | 5.35^***^ | 4.42^***^ | 4.54^***^ | 1.49^***^ | 1.05^*^ | 1.59^***^ | 3.43^***^ | 3.68^***^ | 4.16^***^ |
| GN08 | 3.18^***^ | 7.32^***^ | 5.61^***^ | 4.04^***^ | 4.79^***^ | 4.88^***^ | 3.96^***^ | 4.07^***^ | 1.02^*^ | 0.58^NS^ | 1.13^*^ | 2.96^***^ | 3.22^***^ | 3.69^***^ |
| GN09 | 1.46^***^ | 5.61^***^ | 3.90^***^ | 2.32^***^ | 3.07^***^ | 3.17^***^ | 2.24^***^ | 2.35^***^ | 0.69^NS^ | 1.14^*^ | 0.59^NS^ | 1.24^**^ | 1.50^***^ | 1.97^***^ |
| GN10 | 2.15^***^ | 6.30^***^ | 4.59^***^ | 3.01^***^ | 3.76^***^ | 3.86^***^ | 2.93^***^ | 3.04^***^ | 0.01^NS^ | 0.45^NS^ | 0.10^NS^ | 1.93^***^ | 2.19^***^ | 2.66^***^ |
| GN11 | 6.04^***^ | 1.89^***^ | 3.60^***^ | 5.18^***^ | 4.43^***^ | 4.33^***^ | 5.26^***^ | 5.15^***^ | 8.19^***^ | 8.64^***^ | 8.09^***^ | 6.26^***^ | 6.00^***^ | 5.23^***^ |
| GN12 | 1.89^***^ | 2.25^***^ | 0.54^**^ | 1.03^*^ | 0.28^NS^ | 0.19^NS^ | 1.11^*^ | 1.00^*^ | 4.05^***^ | 4.49^***^ | 3.94^***^ | 2.11^***^ | 1.85^***^ | 1.38^**^ |
| GN13 | 3.55^***^ | 0.60^NS^ | 1.11^*^ | 2.69^***^ | 1.94^***^ | 1.84^***^ | 2.77^***^ | 2.66^***^ | 5.70^***^ | 6.15^***^ | 5.60^***^ | 3.77^***^ | 3.51^***^ | 3.04^***^ |
| GN14 | 0.01^NS^ | 4.13^***^ | 2.42^***^ | 0.85^NS^ | 1.59^***^ | 1.69^***^ | 0.76^**^ | 0.88^NS^ | 2.17^***^ | 2.62^***^ | 2.07^***^ | 0.24^NS^ | 0.02^NS^ | 0.49^NS^ |
| GN15 | ---- | 4.14^***^ | 2.43^***^ | 0.86^NS^ | 1.61^***^ | 1.71^***^ | 0.78^NS^ | 0.89^*^ | 2.16^***^ | 2.60^***^ | 2.05^***^ | 0.22^NS^ | 0.04^NS^ | 0.51^NS^ |
| GN16 | 3.88^***^ | ---- | 1.71^***^ | 3.28^***^ | 2.54^***^ | 2.44^***^ | 3.38^***^ | 3.25^***^ | 6.30^***^ | 6.75^***^ | 6.20^***^ | 4.37^***^ | 4.11^***^ | 3.64^***^ |
| GN17 | 1.71^***^ | 2.17^***^ | ---- | 1.57^***^ | 0.83^NS^ | 0.73^NS^ | 1.66^***^ | 1.54^**^ | 4.59^***^ | 5.04^***^ | 4.49^***^ | 2.66^***^ | 2.40^***^ | 1.93^***^ |
| GN18 | 1.00^NS^ | 2.88^***^ | 0.71^NS^ | ---- | 0.75^NS^ | 0.85^NS^ | 0.08^NS^ | 0.03^NS^ | 3.02^***^ | 3.46^***^ | 2.91^***^ | 1.08^*^ | 0.82^NS^ | 0.35^NS^ |
| GN19 | 1.23^*^ | 2.65^***^ | 0.48^NS^ | 0.23^NS^ | ---- | 0.10^NS^ | 0.83^NS^ | 0.71^NS^ | 3.76^***^ | 4.21^***^ | 3.66^***^ | 1.83^***^ | 1.57^***^ | 1.10^**^ |
| GN20 | 1.26^*^ | 2.62^***^ | 0.45^NS^ | 0.26^NS^ | 0.03^NS^ | ---- | 0.93^*^ | 0.82^NS^ | 3.68^***^ | 4.31^***^ | 3.76^***^ | 1.93^***^ | 1.67^***^ | 1.20^**^ |
| GN21 | 0.22^NS^ | 3.66^***^ | 1.48^**^ | 0.77^NS^ | 1.00^*^ | 1.04^*^ | ---- | 0.11^NS^ | 2.93^***^ | 3.38^***^ | 2.83^***^ | 1.00^*^ | 0.74^NS^ | 0.27^NS^ |
| GN22 | 0.31^NS^ | 4.19^***^ | 2.01^***^ | 1.30^*^ | 1.53^**^ | 1.57^**^ | 0.53^NS^ | ---- | 3.05^***^ | 3.49^***^ | 2.94^***^ | 1.11^*^ | 0.86^NS^ | 0.38^NS^ |
| GN23 | 2.73^***^ | 6.61^***^ | 4.44^***^ | 3.73^***^ | 3.96^***^ | 3.99^***^ | 2.95^***^ | 2.42^***^ | ---- | 0.44^NS^ | 0.11^NS^ | 1.94^***^ | 2.19^***^ | 2.67^***^ |
| GN24 | 2.76^***^ | 6.64^***^ | 4.46^***^ | 3.75^***^ | 3.98^***^ | 4.01^***^ | 2.98^***^ | 2.45^***^ | 0.02^NS^ | ---- | 0.55^NS^ | 2.38^***^ | 2.64^***^ | 3.11^***^ |
| GN25 | 1.81^***^ | 5.69^***^ | 3.52^***^ | 2.81^***^ | 3.04^***^ | 3.07^***^ | 2.04^***^ | 1.51^**^ | 0.92^NS^ | 0.94^NS^ | ---- | 1.83^***^ | 2.09^***^ | 2.56^***^ |
| GN26 | 1.64^***^ | 5.52^***^ | 3.34^***^ | 2.64^***^ | 2.86^***^ | 2.90^***^ | 1.86^***^ | 1.33^**^ | 1.09^*^ | 1.12^*^ | 0.17^**^ | ---- | 0.26^NS^ | 0.73^NS^ |
| GN27 | 1.55^**^ | 5.43^***^ | 3.26^***^ | 2.55^***^ | 2.78^***^ | 2.81^***^ | 1.77^***^ | 1.25^*^ | 1.18^*^ | 1.20^*^ | 0.26^**^ | 0.09^NS^ | ---- | 0.47^NS^ |
| GN28 | 0.11^NS^ | 3.99^***^ | 1.82^***^ | 1.11^*^ | 1.34^**^ | 1.37 | 0.34^NS^ | 0.19^NS^ | 2.62^***^ | 2.64^***^ | 1.70^***^ | 1.53^**^ | 1.44^**^ | ---- |

**Table S3.** Comparison of significant differences in mean stone transverse diameter (below the diagonal) and longitudinal diameter (above the diagonal) of individuals of *Malania oleifera* at Guangnan (LSD test)

| Tree | GN01 | GN02 | GN03 | GN04 | GN05 | GN06 | GN07 | GN08 | GN09 | GN10 | GN11 | GN12 | GN13 | GN14 |
| --- | --- | --- | --- | --- | --- | --- | --- | --- | --- | --- | --- | --- | --- | --- |
| GN01 | ---- | 2.98^**^ | 0.93^*^ | 4.49^**^ | 3.97^**^ | 4.24^**^ | 5.56^**^ | 5.40^**^ | 3.82^**^ | 4.96^**^ | 0.57^NS^ | 3.06^**^ | 2.23^**^ | 4.24^**^ |
| GN02 | 3.61^***^ | ---- | 3.90^**^ | 1.52^**^ | 0.99^*^ | 1.26^**^ | 2.58^**^ | 2.43^**^ | 0.84^*^ | 1.97^**^ | 2.41^**^ | 0.09^NS^ | 0.75^NS^ | 1.26^**^ |
| GN03 | 1.01^*^ | 2.61^***^ | ---- | 5.42^**^ | 4.90^**^ | 5.16^**^ | 6.49^**^ | 6.33^**^ | 4.75^**^ | 5.88^**^ | 1.50^**^ | 3.99^**^ | 3.15^**^ | 5.16^**^ |
| GN04 | 3.75^***^ | 0.14^NS^ | 2.75^***^ | ---- | 0.53^NS^ | 0.26^NS^ | 1.07^**^ | 0.91^*^ | 0.68^NS^ | 0.46^NS^ | 3.93^**^ | 1.43^**^ | 2.27^**^ | 0.26^NS^ |
| GN05 | 2.87^***^ | 0.75^NS^ | 1.86^***^ | 0.89^*^ | ---- | 0.27^NS^ | 1.59^**^ | 1.44^**^ | 0.15^NS^ | 0.99^*^ | 3.40^**^ | 0.91^*^ | 1.74^**^ | 0.27^NS^ |
| GN06 | 1.39^***^ | 2.23^***^ | 0.38^NS^ | 2.37^***^ | 1.48^***^ | ---- | 1.32^**^ | 1.17^**^ | 0.42^NS^ | 0.72^NS^ | 3.67^**^ | 1.18^**^ | 2.01^**^ | 0.00^NS^ |
| GN07 | 2.74^***^ | 0.87^*^ | 1.74^***^ | 1.01^*^ | 0.13^NS^ | 1.35^***^ | ---- | 0.16^NS^ | 1.74^**^ | 0.60^NS^ | 4.99^**^ | 2.50^**^ | 3.33^**^ | 1.32^**^ |
| GN08 | 1.43^***^ | 2.19^***^ | 0.42^NS^ | 2.33^***^ | 1.44^***^ | 0.04^NS^ | 1.31^**^ | ---- | 1.59^**^ | 0.45^NS^ | 4.84^**^ | 2.34^**^ | 3.18^**^ | 1.17^**^ |
| GN09 | 1.83^***^ | 1.78^***^ | 0.82^*^ | 1.92^***^ | 1.04^*^ | 0.44^NS^ | 0.91^*^ | 0.40^**^ | ---- | 1.14^**^ | 3.25^**^ | 0.75^NS^ | 1.59^**^ | 0.42^NS^ |
| GN10 | 2.55^***^ | 1.07^*^ | 1.54^***^ | 1.21^**^ | 0.32^NS^ | 1.16^**^ | 0.19^NS^ | 1.12^**^ | 0.72^NS^ | ---- | 4.39^**^ | 1.89^**^ | 2.73^**^ | 0.72^NS^ |
| GN11 | 2.03^***^ | 5.64^***^ | 3.04^***^ | 5.79^***^ | 4.90^***^ | 3.42^***^ | 4.77^***^ | 3.46^***^ | 3.86^***^ | 4.58^***^ | ---- | 2.50^**^ | 1.66^**^ | 3.67^**^ |
| GN12 | 0.20^**^ | 3.81^***^ | 1.20^**^ | 3.95^***^ | 3.07^***^ | 1.59^***^ | 2.94^***^ | 1.63^***^ | 2.03^***^ | 2.75^***^ | 1.83^***^ | ---- | 0.84^*^ | 1.17^**^ |
| GN13 | 0.51^NS^ | 4.12^***^ | 1.51^***^ | 4.26^***^ | 3.38^***^ | 1.90^***^ | 3.25^***^ | 1.94^***^ | 2.34^***^ | 3.06^***^ | 1.52^***^ | 0.31^NS^ | ---- | 2.01^**^ |
| GN14 | 1.75^***^ | 1.86^***^ | 0.75^NS^ | 2.00^***^ | 1.12^**^ | 0.36^NS^ | 0.99^*^ | 0.33^NS^ | 0.08^NS^ | 0.80^NS^ | 3.78^***^ | 1.95^***^ | 2.26^***^ | ---- |
| GN15 | 0.84^*^ | 2.78^***^ | 0.17^NS^ | 2.92^***^ | 2.03^***^ | 0.55^NS^ | 1.90^***^ | 0.59^NS^ | 0.99^*^ | 1.71^***^ | 2.87^***^ | 1.04^*^ | 1.35^***^ | 0.92^*^ |
| GN16 | 0.51^NS^ | 4.12^***^ | 1.51^***^ | 4.26^***^ | 3.38^***^ | 1.89^***^ | 3.25^***^ | 1.93^***^ | 2.34^***^ | 3.05^***^ | 1.52^***^ | 0.31^NS^ | 0.00^NS^ | 2.26^***^ |
| GN17 | 0.48^NS^ | 3.13^***^ | 0.52^NS^ | 3.27^***^ | 2.39^***^ | 0.91^*^ | 2.26^***^ | 0.95^*^ | 1.35^***^ | 2.06^***^ | 2.51^***^ | 0.68^NS^ | 0..99^*^ | 1.27^**^ |
| GN18 | 0.82^*^ | 2.79^***^ | 0.18^NS^ | 2.93^***^ | 2.05^***^ | 0.57^NS^ | 1.92^***^ | 0.61^NS^ | 1.01^*^ | 1.73^***^ | 2.85^***^ | 1.02^*^ | 1.33^**^ | 0.93^*^ |
| GN19 | 0.66^NS^ | 2.96^***^ | 0.35^NS^ | 3.10^***^ | 2.21^***^ | 0.73^NS^ | 2.08^***^ | 0.77^NS^ | 1.17^**^ | 1.89^***^ | 2.69^***^ | 0.86^NS^ | 1.17^**^ | 1.09^**^ |
| GN20 | 0.76^NS^ | 2.85^***^ | 0.25^NS^ | 2.99^***^ | 2.11^***^ | 0.63^NS^ | 1.98^***^ | 0.67^NS^ | 1.07^*^ | 1.78^***^ | 2.79^***^ | 0.86^*^ | 1.27^**^ | 0.99^*^ |
| GN21 | 0.18^NS^ | 3.43^***^ | 0.83^NS^ | 3.57^***^ | 2.69^***^ | 1.21^**^ | 2.56^***^ | 1.25^**^ | 1.65^***^ | 2.37^***^ | 2.21^***^ | 0.38^NS^ | 0.69^NS^ | 1.57^***^ |
| GN22 | 0.33^NS^ | 3.28^***^ | 0.67^NS^ | 3.42^***^ | 2.53^***^ | 1.05^*^ | 2.41^***^ | 1.09^**^ | 1.50^***^ | 2.21^***^ | 2.37^***^ | 0.53^NS^ | 0.84^*^ | 1.42^***^ |
| GN23 | 2.35^***^ | 1.27^**^ | 1.34^***^ | 1.41^***^ | 0.52^NS^ | 0.96^*^ | 0.40^NS^ | 0.92^*^ | 0.52^NS^ | 0.20^NS^ | 4.38^***^ | 2.54^***^ | 2.85^***^ | 0.59^NS^ |
| GN24 | 2.10^***^ | 1.51^***^ | 1.09^**^ | 1.65^***^ | 0.77^NS^ | 0.71^NS^ | 0.64^NS^ | 0.67^NS^ | 0.27^NS^ | 0.45^NS^ | 4.13^***^ | 2.30^***^ | 2.61^***^ | 0.35^NS^ |
| GN25 | 1.50^***^ | 2.12^***^ | 0.49^NS^ | 2.26^***^ | 1.37^***^ | 0.11^NS^ | 1.24^**^ | 0.07^NS^ | 0.33^NS^ | 1.05^*^ | 3.53^***^ | 1.70^***^ | 2.01^***^ | 0.26^NS^ |
| GN26 | 1.84^***^ | 1.78^***^ | 0.83^*^ | 1.91^***^ | 1.03^*^ | 0.45^NS^ | 0.90^*^ | 0.41^NS^ | 0.01^NS^ | 0.71^NS^ | 3.87^***^ | 2.04^***^ | 2.35^***^ | 0.08^NS^ |
| GN27 | 2.21^***^ | 1.40^***^ | 1.21^**^ | 1.54^**^ | 0.66^NS^ | 0.83^*^ | 0.50^NS^ | 0.78^NS^ | 0.38^NS^ | 0.33^NS^ | 4.24^***^ | 2.41^***^ | 2.72^***^ | 0.46^NS^ |
| GN28 | 1.38^***^ | 2.34^***^ | 0.37^NS^ | 2.38^**^ | 1.49^***^ | 0.01^NS^ | 1.36^***^ | 0.05^NS^ | 0.45^NS^ | 1.17^**^ | 3.41^***^ | 1.58^***^ | 1.89^***^ | 0.38^NS^ |

NS *p* > 0.05, * *p* ≤ 0.05, ** *p* ≤ 0.01, *** *p* ≤ 0.001

**Table S3.** (continued)

| Tree | GN15 | GN16 | GN17 | GN18 | GN19 | GN20 | GN21 | GN22 | GN23 | GN24 | GN25 | GN26 | GN27 | GN28 |
| --- | --- | --- | --- | --- | --- | --- | --- | --- | --- | --- | --- | --- | --- | --- |
| GN01 | 2.97^***^ | 1.67^***^ | 2.40^***^ | 3.32^***^ | 2.66^***^ | 2.82^***^ | 1.97^***^ | 2.08^***^ | 4.20^***^ | 1.80^***^ | 1.50^***^ | 1.09^**^ | 0.59^NS^ | 0.75^NS^ |
| GN02 | 0.00^NS^ | 1.30^***^ | 0.58^NS^ | 0.35^NS^ | 0.31^NS^ | 0.16^NS^ | 1.00^**^ | 0.90^*^ | 1.22^**^ | 1.17^**^ | 1.48^***^ | 1.88^***^ | 2.39^***^ | 2.23^***^ |
| GN03 | 3.90^***^ | 2.60^***^ | 3.32^***^ | 4.25^***^ | 3.59^***^ | 3.74^***^ | 2.90^***^ | 3.01^***^ | 5.13^***^ | 2.73^***^ | 2.43^***^ | 2.02^***^ | 1.52^***^ | 1.68^***^ |
| GN04 | 1.52^***^ | 2.82^***^ | 2.09^***^ | 1.17^**^ | 1.83^***^ | 1.68^***^ | 2.52^***^ | 2.42^***^ | 0.30^NS^ | 2.69^***^ | 3.00^***^ | 3.40^***^ | 3.90^***^ | 3.74^***^ |
| GN05 | 1.00^**^ | 2.30^***^ | 1.57^***^ | 0.65^NS^ | 1.30^***^ | 1.15^**^ | 2.00^***^ | 1.89^***^ | 0.23^NS^ | 2.17^***^ | 2.47^***^ | 2.87^***^ | 3.39^***^ | 3.22^***^ |
| GN06 | 1.27^***^ | 2.57^***^ | 1.84^***^ | 0.92^*^ | 1.57^***^ | 1.42^***^ | 2.26^***^ | 2.16^***^ | 0.04^NS^ | 2.44^***^ | 2.74^***^ | 3.14^***^ | 3.65^***^ | 3.49^***^ |
| GN07 | 2.59^***^ | 3.89^***^ | 3.16^***^ | 2.24^***^ | 2.89^***^ | 2.74^***^ | 3.59^***^ | 3.48^***^ | 1.36^***^ | 3.76^***^ | 4.06^***^ | 4.47^***^ | 4.97^***^ | 4.81^***^ |
| GN08 | 2.43^***^ | 3.73^***^ | 3.00^***^ | 2.08^***^ | 2.74^***^ | 2.59^***^ | 3.43^***^ | 3.33^***^ | 1.21^**^ | 3.60^***^ | 3.91^***^ | 4.31^***^ | 4.81^***^ | 4.65^***^ |
| GN09 | 0.85^*^ | 2.15^***^ | 1.42^***^ | 0.50^NS^ | 1.15^**^ | 1.00^**^ | 1.84^***^ | 1.74^***^ | 0.38^NS^ | 2.01^***^ | 2.32^***^ | 2.72^***^ | 3.23^***^ | 3.07^***^ |
| GN10 | 1.98^***^ | 3.28^***^ | 2.56^***^ | 1.63^***^ | 2.29^***^ | 2.14^***^ | 2.98^***^ | 2.88^***^ | 0.76^NS^ | 3.15^***^ | 3.45^***^ | 3.86^***^ | 4.37^***^ | 4.21^***^ |
| GN11 | 2.40^***^ | 1.10^**^ | 1.83^***^ | 2.76^***^ | 2.10^***^ | 2.25^***^ | 1.41^***^ | 1.51^***^ | 3.63^***^ | 1.23^**^ | 0.93^*^ | 0.53^NS^ | 0.02^NS^ | 0.18^NS^ |
| GN12 | 0.09^NS^ | 1.39^***^ | 0.66^NS^ | 0.26^NS^ | 0.40^NS^ | 0.25^NS^ | 1.09^**^ | 0.98^*^ | 1.14^**^ | 1.26^**^ | 1.57^***^ | 1.97^***^ | 2.47^***^ | 2.31^***^ |
| GN13 | 0.75^NS^ | 0.55^NS^ | 0.17^NS^ | 1.10^**^ | 0.44^NS^ | 0.59^NS^ | 0.25^NS^ | 0.15^NS^ | 1.97^***^ | 0.42^NS^ | 0.73^NS^ | 1.13^**^ | 1.64^***^ | 1.48^***^ |
| GN14 | 1.26^***^ | 2.56^***^ | 1.84^***^ | 0.91^*^ | 1.57^***^ | 1.42^***^ | 2.26^***^ | 2.16^***^ | 0.04^NS^ | 2.43^***^ | 2.74^***^ | 3.14^***^ | 3.65^***^ | 3.49^***^ |
| GN15 | ---- | 1.30^***^ | 0.57^NS^ | 0.35^NS^ | 0.31^NS^ | 0.16^NS^ | 1.00^**^ | 0.89^*^ | 1.23^**^ | 1.17^**^ | 1.47^***^ | 1.88^***^ | 2.38^***^ | 2.22^***^ |
| GN16 | 1.34^***^ | ---- | 0.73^NS^ | 1.65^***^ | 0.99^**^ | 1.14^**^ | 0.30^NS^ | 0.41^NS^ | 2.53^***^ | 0.13^NS^ | 0.17^NS^ | 0.58^NS^ | 1.08^**^ | 0.92^*^ |
| GN17 | 0.35^NS^ | 0.99^*^ | ---- | 0.92^*^ | 0.26^NS^ | 0.42^NS^ | 0.43^NS^ | 0.32^NS^ | 1.80^***^ | 0.60^NS^ | 0.90^*^ | 1.31^***^ | 1.81^***^ | 1.65^***^ |
| GN18 | 0.02^NS^ | 1.33^**^ | 0.34^NS^ | ---- | 0.66^NS^ | 0.51^NS^ | 1.35^***^ | 1.24^***^ | 0.88^*^ | 1.52^***^ | 1.82^***^ | 2.23^***^ | 2.73^***^ | 2.57^***^ |
| GN19 | 0.18^NS^ | 1.16^**^ | 0.18^NS^ | 0.16^NS^ | ---- | 1.52^***^ | 0.69^NS^ | 0.59^NS^ | 1.53^***^ | 0.86^*^ | 1.17^**^ | 1.57^***^ | 2.07^***^ | 1.92^***^ |
| GN20 | 0.08^NS^ | 1.27^**^ | 0.28^NS^ | 0.06^NS^ | 0.10^NS^ | ---- | 0.84^*^ | 0.74^NS^ | 1.38^***^ | 1.01^**^ | 1.32^***^ | 1.72^***^ | 2.23^***^ | 2.07^***^ |
| GN21 | 0.66^NS^ | 0.69^NS^ | 0.30^NS^ | 0.64^NS^ | 0.48^NS^ | 0.58^NS^ | ---- | 0.11^NS^ | 2.22^***^ | 0.17^NS^ | 0.48^NS^ | 0.88^*^ | 1.38^***^ | 1.22^**^ |
| GN22 | 0.50^NS^ | 0.84^*^ | 0.15^NS^ | 0.49^NS^ | 0.32^NS^ | 0.43^NS^ | 0.15^NS^ | ---- | 2.12^***^ | 0.28^NS^ | 0.58^NS^ | 0.99^*^ | 1.49^***^ | 1.33^***^ |
| GN23 | 1.51^***^ | 2.85^***^ | 1.86^***^ | 1.52^***^ | 1.69^***^ | 1.59^***^ | 2.17^***^ | 2.01^***^ | ---- | 2.40^***^ | 2.70^***^ | 3.10^***^ | 3.61^***^ | 3.45^***^ |
| GN24 | 1.26^**^ | 2.61^***^ | 1.62^***^ | 1.28^**^ | 1.44^***^ | 1.34^***^ | 1.92^***^ | 1.77^***^ | 0.25^NS^ | ---- | 0.30^NS^ | 0.71^NS^ | 1.21^**^ | 1.05^**^ |
| GN25 | 0.66^NS^ | 2.00^***^ | 1.01^*^ | 0.68^NS^ | 0.84^*^ | 0.74^**^ | 1.32^**^ | 1.16^**^ | 0.85^*^ | 0.60^NS^ | ---- | 0.40^NS^ | 0.91^*^ | 0.75^NS^ |
| GN26 | 1.00^*^ | 2.34^***^ | 1.35^***^ | 1.02^*^ | 1.18^**^ | 1.08^**^ | 1.66^***^ | 1.50^****^ | 0.51^NS^ | 0.26^NS^ | 0.34^NS^ | ---- | 0.50^NS^ | 0.34^NS^ |
| GN27 | 1.38^***^ | 2.72^***^ | 1.73^***^ | 1.39^***^ | 1.56^***^ | 1.45^***^ | 2.03^***^ | 1.88^***^ | 0.13^NS^ | 0.11^NS^ | 0.72^NS^ | 0.38^NS^ | ---- | 0.16^NS^ |
| GN28 | 0.54^**^ | 1.88^***^ | 0.89^*^ | 0.56^NS^ | 0.72^NS^ | 0.62^NS^ | 1.20^**^ | 1.04^*^ | 0.97^*^ | 0.72^NS^ | 0.12^NS^ | 0.46^NS^ | 0.84^*^ | ---- |

**Table S4.** Comparison of significant differences in mean weight of fruit (below the diagonal) and stone (above the diagonal) from individuals of *Malania oleifera* in Funing (LSD test)

| Tree | FN01 | FN02 | FN03 | FN04 | FN05 | FN06 | FN07 | FN08 | FN09 | FN10 | FN11 | FN12 | FN13 |
| --- | --- | --- | --- | --- | --- | --- | --- | --- | --- | --- | --- | --- | --- |
| FN01 | ---- | 1.52^***^ | 1.24^**^ | 0.33 ^NS^ | 2.78^***^ | 2.93^***^ | 3.31^***^ | 5.69^***^ | 4.26^***^ | 1.38^**^ | 2.44^***^ | 1.69^***^ | 1.10^*^ |
| FN02 | 3.34^**^ | ---- | 0.29^NS^ | 1.19^*^ | 1.25^*^ | 1.40^**^ | 4.83^***^ | 4.16^***^ | 2.74^***^ | 0.14^NS^ | 0.91^NS^ | 3.21^***^ | 0.42^NS^ |
| FN03 | 8.76^***^ | 5.41^***^ | ---- | 0.91^NS^ | 1.54^***^ | 1.69^***^ | 4.55^***^ | 4.45^***^ | 3.03^***^ | 0.14^NS^ | 1.20^**^ | 2.92^***^ | 0.13^NS^ |
| FN04 | 6.36^***^ | 3.02^*^ | 2.39^**^ | ---- | 2.45^***^ | 2.60^***^ | 3.64^***^ | 5.36^***^ | 3.93^***^ | 1.05^*^ | 2.10^***^ | 2.02^***^ | 0.78^NS^ |
| FN05 | 18.73^***^ | 15.39^***^ | 9.97^***^ | 12.37^***^ | ---- | 0.15^NS^ | 6.09^***^ | 2.91^***^ | 1.49^***^ | 1.40^**^ | 0.34^NS^ | 4.46^***^ | 1.67^***^ |
| FN06 | 3.99^***^ | 0.65^NS^ | 4.76^***^ | 2.37^*^ | 14.73^***^ | ---- | 6.23^***^ | 2.76^***^ | 1.34^**^ | 1.55^**^ | 0.49^NS^ | 4.61^***^ | 1.82^***^ |
| FN07 | 4.39^***^ | 7.74^***^ | 13.15^***^ | 10.76^***^ | 23.12^***^ | 8.39^***^ | ---- | 9.00^***^ | 7.57^***^ | 4.69^***^ | 5.74^***^ | 1.62^***^ | 4.41^***^ |
| FN08 | 18.88^***^ | 15.54^***^ | 10.12^***^ | 12.52^***^ | 0.16^NS^ | 14.89^***^ | 23.28^***^ | ---- | 1.42^**^ | 4.31^***^ | 3.25^***^ | 7.37^***^ | 4.58^***^ |
| FN09 | 18.36^***^ | 15.02^***^ | 9.61^***^ | 12.00^***^ | 0.37^NS^ | 14.37^***^ | 22.76^***^ | 0.52^NS^ | ---- | 2.89^***^ | 1.83^***^ | 5.95^***^ | 3.16^***^ |
| FN10 | 9.89^***^ | 6.55^***^ | 1.14^NS^ | 3.53^**^ | 8.83^***^ | 5.90^***^ | 14.29^***^ | 8.99^***^ | 8.47^***^ | ---- | 1.06^*^ | 3.06^***^ | 0.27^NS^ |
| FN11 | 1.44^NS^ | 1.90^NS^ | 7.31^***^ | 4.92^***^ | 17.29^***^ | 2.55^**^ | 5.84^***^ | 17.44^***^ | 16.92^***^ | 8.45^***^ | ---- | 4.12^***^ | 1.33^**^ |
| FN12 | 9.02^***^ | 5.68^***^ | 0.27^NS^ | 2.66^*^ | 9.71^***^ | 5.03^***^ | 13.42^***^ | 9.86^***^ | 9.34^***^ | 0.87^NS^ | 7.58^***^ | ---- | 2.79^***^ |
| FN13 | 10.63^***^ | 7.29^***^ | 1.87^NS^ | 4.27^***^ | 8.10^***^ | 6.63^***^ | 15.02^***^ | 8.25^***^ | 7.73^***^ | 0.74^NS^ | 9.19^***^ | 1.61^NS^ | ---- |
| FN14 | 11.10^***^ | 7.76^***^ | 2.35^*^ | 4.75^***^ | 7.62^***^ | 7.11^***^ | 15.50^***^ | 7.78^***^ | 7.26^***^ | 1.21^NS^ | 9.66^***^ | 2.08^NS^ | 0.48^NS^ |
| FN15 | 19.80^***^ | 16.46^***^ | 11.05^***^ | 13.44^***^ | 1.07^NS^ | 15.81^***^ | 24.20^***^ | 0.92^NS^ | 1.44^NS^ | 9.91^***^ | 18.36^***^ | 10.78^***^ | 9.17^***^ |
| FN16 | 12.40^***^ | 9.06^***^ | 3.65^**^ | 6.04^***^ | 6.33^***^ | 8.41^***^ | 16.80^***^ | 6.48^***^ | 5.96^***^ | 2.51^*^ | 10.96^***^ | 3.38^**^ | 1.77^NS^ |
| FN17 | 6.30^***^ | 2.95^*^ | 2.46^*^ | 0.07^NS^ | 12.43^***^ | 2.30^*^ | 10.69^***^ | 12.59^***^ | 12.07^***^ | 3.60^**^ | 4.85^***^ | 2.73^*^ | 4.33^***^ |
| FN18 | 17.20^***^ | 20.54^***^ | 25.95^***^ | 23.56^***^ | 35.93^***^ | 21.19^***^ | 12.80^***^ | 36.08^***^ | 35.56^***^ | 27.09^***^ | 18.64^***^ | 26.22^***^ | 27.83^***^ |
| FN19 | 8.53^***^ | 5.19^***^ | 0.23^NS^ | 2.17^NS^ | 10.20^***^ | 4.53^***^ | 12.92^***^ | 10.35^***^ | 9.83^***^ | 1.36^NS^ | 7.09^***^ | 0.49^NS^ | 2.10^NS^ |
| FN20 | 6.25^***^ | 9.59^***^ | 15.01^***^ | 12.61^***^ | 24.98^***^ | 10.24^***^ | 1.86^NS^ | 25.13^***^ | 24.61^***^ | 16.15^***^ | 7.69^***^ | 15.27^***^ | 16.88^***^ |
| FN21 | 8.12^***^ | 11.47^***^ | 16.88^***^ | 14.49^***^ | 26.85^***^ | 12.12^***^ | 3.73^**^ | 27.01^***^ | 26.49^***^ | 18.02^***^ | 9.57^***^ | 17.15^***^ | 18.75^***^ |
| FN22 | 5.09^***^ | 1.75^NS^ | 3.67^**^ | 1.27^NS^ | 13.64^***^ | 1.10^NS^ | 9.48^***^ | 13.79^***^ | 13.27^***^ | 4.80^***^ | 3.65^**^ | 3.93^***^ | 5.54^***^ |
| FN23 | 6.99^***^ | 3.65^**^ | 1.76^NS^ | 0.63^NS^ | 11.74^***^ | 3.00^*^ | 11.39^***^ | 11.89^***^ | 11.37^***^ | 2.90^*^ | 5.55^***^ | 2.03^NS^ | 3.64^**^ |
| FN24 | 5.44^***^ | 2.10^NS^ | 3.32^**^ | 0.92^NS^ | 13.29^***^ | 1.45^NS^ | 9.83^***^ | 13.44^***^ | 12.92^***^ | 4.45^***^ | 4.00^***^ | 3.58^**^ | 5.19^***^ |
| FN25 | 4.77^***^ | 1.42^NS^ | 3.99^**^ | 1.60^NS^ | 13.96^***^ | 0.77^NS^ | 9.16^***^ | 14.12^***^ | 13.60^***^ | 5.13^***^ | 3.32^**^ | 4.26^***^ | 5.86^***^ |

NS *p* > 0.05, * *p* ≤ 0.05, ** *p* ≤ 0.01, *** *p* ≤ 0.001

**Table S4.** (continued)

| Tree | FN14 | FN15 | FN16 | FN17 | FN18 | FN19 | FN20 | FN21 | FN22 | FN23 | FN24 | FN25 |
| --- | --- | --- | --- | --- | --- | --- | --- | --- | --- | --- | --- | --- |
| FN01 | 1.31^**^ | 6.74^***^ | 4.44^***^ | 4.48^***^ | 3.91^***^ | 1.90^***^ | 4.60^***^ | 1.52^***^ | 1.58^***^ | 2.62^***^ | 0.67^NS^ | 1.26^**^ |
| FN02 | 0.21^NS^ | 5.21^***^ | 2.91^***^ | 2.96^***^ | 5.43^***^ | 0.38^NS^ | 6.12^***^ | 3.04^***^ | 0.06^NS^ | 1.10^*^ | 0.85^NS^ | 0.26^NS^ |
| FN03 | 0.07^NS^ | 5.50^***^ | 3.20^***^ | 3.24^***^ | 5.15^***^ | 0.66^NS^ | 5.84^***^ | 2.76^***^ | 0.35^NS^ | 1.38^**^ | 0.57^NS^ | 0.03^NS^ |
| FN04 | 0.98^*^ | 6.41^***^ | 4.11^***^ | 4.15^***^ | 4.24^***^ | 1.57^***^ | 4.92^***^ | 1.85^***^ | 1.26^**^ | 2.29^***^ | 0.34^NS^ | 0.94^*^ |
| FN05 | 1.47^**^ | 3.96^***^ | 1.66^***^ | 1.70^***^ | 6.69^***^ | 0.88^NS^ | 7.38^***^ | 4.30^***^ | 1.19^**^ | 0.16^NS^ | 2.11^***^ | 1.51^***^ |
| FN06 | 1.62^***^ | 3.81^***^ | 1.51^***^ | 1.55^***^ | 6.83^***^ | 1.03^*^ | 7.53^***^ | 4.44^***^ | 1.34^**^ | 0.31^NS^ | 2.26^***^ | 1.66^***^ |
| FN07 | 4.62^***^ | 10.05^***^ | 7.74^***^ | 7.79^***^ | 0.60^NS^ | 5.21^***^ | 1.29^**^ | 1.79^***^ | 4.89^***^ | 5.93^***^ | 3.98^***^ | 4.57^***^ |
| FN08 | 4.38^***^ | 1.05^*^ | 1.25^**^ | 1.21^**^ | 9.60^***^ | 3.79^***^ | 10.29^***^ | 7.21^***^ | 4.10^***^ | 3.07^***^ | 5.02^***^ | 4.42^***^ |
| FN09 | 2.96^***^ | 2.47^***^ | 0.17^NS^ | 0.22^NS^ | 8.17^***^ | 2.36^***^ | 8.86^***^ | 5.78^***^ | 2.68^***^ | 1.64^***^ | 3.59^***^ | 3.00^***^ |
| FN10 | 0.07^NS^ | 5.36^***^ | 3.06^***^ | 3.10^***^ | 5.29^***^ | 0.52^**^ | 5.98^***^ | 2.90^***^ | 0.21^NS^ | 1.24^**^ | 0.71^NS^ | 0.11^NS^ |
| FN11 | 1.13^*^ | 4.30^***^ | 2.00^***^ | 2.04^***^ | 6.34^***^ | 0.54^NS^ | 7.04^***^ | 3.95^***^ | 0.85^NS^ | 0.18^NS^ | 1.77^***^ | 1.17^*^ |
| FN12 | 2.99^***^ | 8.42^***^ | 6.12^***^ | 6.17^***^ | 2.22^***^ | 3.59^***^ | 2.91^***^ | 0.17^NS^ | 3.27^***^ | 4.31^***^ | 2.36^***^ | 2.95^***^ |
| FN13 | 0.20^NS^ | 5.63^***^ | 3.33^***^ | 3.37^***^ | 5.01^***^ | 0.80^NS^ | 5.70^***^ | 2.62^***^ | 0.48^NS^ | 1.52^***^ | 0.44^NS^ | 0.16^NS^ |
| FN14 | ---- | 5.43^***^ | 3.13^***^ | 3.17^***^ | 5.22^***^ | 0.59^NS^ | 5.91^***^ | 2.83^***^ | 0.28^NS^ | 1.31^**^ | 0.64^NS^ | 0.04^NS^ |
| FN15 | 8.70^***^ | ---- | 2.30^***^ | 2.26^***^ | 10.65^***^ | 4.84^***^ | 11.34^***^ | 8.26^***^ | 5.15^***^ | 4.12^***^ | 6.07^***^ | 5.47^***^ |
| FN16 | 1.30^**^ | 7.40^***^ | ---- | 0.04^NS^ | 8.34^***^ | 2.54^***^ | 9.04^***^ | 5.95^***^ | 2.85^***^ | 1.82^***^ | 3.77^***^ | 3.17^***^ |
| FN17 | 4.81^***^ | 13.51^***^ | 6.11^***^ | ---- | 8.39^***^ | 2.58^***^ | 9.08^***^ | 6.00^***^ | 2.90^***^ | 1.86^***^ | 3.81^***^ | 3.21^***^ |
| FN18 | 28.31^***^ | 37.00^***^ | 29.60^***^ | 23.49^***^ | ---- | 5.81^***^ | 0.69^NS^ | 2.39^***^ | 5.49^***^ | 6.53^***^ | 4.58^***^ | 5.17^***^ |
| FN19 | 2.58^**^ | 11.27^***^ | 3.87^***^ | 2.23^NS^ | 25.73^***^ | ---- | 6.50^***^ | 3.42^***^ | 0.32^NS^ | 0.72^NS^ | 1.23^**^ | 0.64^NS^ |
| FN20 | 17.36^***^ | 26.05^***^ | 18.65^***^ | 12.55^***^ | 10.95^***^ | 14.78^***^ | ---- | 3.08^***^ | 6.18^***^ | 7.22^***^ | 5.27^***^ | 5.86^***^ |
| FN21 | 19.23^***^ | 27.92^***^ | 20.53^***^ | 14.42^***^ | 9.07^***^ | 16.65^***^ | 1.87^NS^ | ---- | 3.10^***^ | 4.14^***^ | 2.19^***^ | 2.78^***^ |
| FN22 | 6.02^***^ | 14.71^***^ | 7.31^***^ | 1.20^NS^ | 22.29^***^ | 3.44^**^ | 11.34^***^ | 13.21^***^ | ---- | 1.04^*^ | 0.92^*^ | 0.32^NS^ |
| FN23 | 4.11^***^ | 12.81^***^ | 5.41^***^ | 0.70^NS^ | 24.19^***^ | 1.54^NS^ | 13.24^***^ | 15.12^***^ | 1.90^NS^ | ---- | 1.95^***^ | 1.36^**^ |
| FN24 | 5.67^***^ | 14.36^***^ | 6.96^***^ | 0.85^NS^ | 22.64^***^ | 3.09^**^ | 11.69^***^ | 13.56^***^ | 0.35^NS^ | 1.55^NS^ | ---- | 0.60^NS^ |
| FN25 | 6.34^***^ | 15.04^***^ | 7.64^***^ | 1.53^NS^ | 21.97^***^ | 3.76^**^ | 11.02^***^ | 12.89^***^ | 0.32^NS^ | 2.23^NS^ | 0.67^NS^ | ---- |

**Table S5.** Comparison of significant differences in mean fruit transverse diameter (below the diagonal) and longitudinal diameter (above the diagonal) from individuals of *Malania oleifera* in Funing (LSD test)

| Tree | FN01 | FN02 | FN03 | FN04 | FN05 | FN06 | FN07 | FN08 | FN09 | FN10 | FN11 | FN12 | FN13 |
| --- | --- | --- | --- | --- | --- | --- | --- | --- | --- | --- | --- | --- | --- |
| FN01 | ---- | 1.53^**^ | 5.83^***^ | 4.75^***^ | 4.63^***^ | 0.13^NS^ | 1.24^**^ | 5.08^***^ | 4.76^***^ | 2.89^***^ | 0.83^NS^ | 1.90^***^ | 2.37^***^ |
| FN02 | 2.03^***^ | ---- | 4.30^***^ | 3.22^***^ | 3.10^***^ | 1.41^**^ | 2.77^***^ | 3.55^***^ | 3.23^***^ | 1.36^**^ | 0.70^NS^ | 0.37^NS^ | 0.84^NS^ |
| FN03 | 5.45^***^ | 3.41^***^ | ---- | 1.08^*^ | 1.20^**^ | 5.71^***^ | 7.07^***^ | 0.75^NS^ | 1.07^*^ | 2.94^***^ | 5.00^***^ | 3.93^***^ | 3.46^***^ |
| FN04 | 3.40^***^ | 1.37^*^ | 2.05^***^ | ---- | 0.12^NS^ | 4.62^***^ | 5.97^***^ | 0.34^NS^ | 0.01^NS^ | 1.85^***^ | 3.92^***^ | 2.85^***^ | 2.38^***^ |
| FN05 | 8.10^***^ | 6.07^***^ | 2.65^***^ | 4.70^***^ | ---- | 4.50^***^ | 5.87^***^ | 0.46^NS^ | 0.13^NS^ | 1.73^***^ | 3.80^***^ | 2.73^***^ | 2.26^***^ |
| FN06 | 0.35^NS^ | 2.38^***^ | 5.79^***^ | 3.75^***^ | 8.45^***^ | ---- | 1.37^**^ | 4.96^***^ | 4.63^***^ | 2.77^***^ | 0.70^NS^ | 1.77^***^ | 2.24^***^ |
| FN07 | 1.72^**^ | 3.75^***^ | 7.17^***^ | 5.12^***^ | 9.82^***^ | 1.37^*^ | ---- | 6.32^*^ | 6.00^***^ | 4.13^***^ | 2.07^***^ | 3.14^***^ | 3.61^***^ |
| FN08 | 8.44^***^ | 6.41^***^ | 2.99^***^ | 5.04^***^ | 0.33^NS^ | 8.78^***^ | 10.15^***^ | ---- | 0.32^NS^ | 2.19^***^ | 4.25^***^ | 3.18^***^ | 2.72^***^ |
| FN09 | 7.68^***^ | 5.66^***^ | 2.24^***^ | 4.29^***^ | 0.41^NS^ | 8.03^***^ | 9.41^***^ | 0.75^NS^ | ---- | 1.87^***^ | 3.93^***^ | 2.86^***^ | 2.39^***^ |
| FN10 | 3.32^***^ | 1.29^*^ | 2.13^***^ | 0.08^NS^ | 4.79^***^ | 3.66^***^ | 5.04^***^ | 5.12^***^ | 4.37^***^ | ---- | 2.06^***^ | 0.99^*^ | 0.53^NS^ |
| FN11 | 2.64^***^ | 0.61^NS^ | 2.81^***^ | 0.76^NS^ | 5.46^***^ | 2.99^***^ | 4.36^***^ | 5.80^***^ | 5.05^***^ | 0.68^NS^ | ---- | 1.07^*^ | 1.54^***^ |
| FN12 | 3.02^***^ | 0.99^NS^ | 2.43^***^ | 0.38^NS^ | 5.08^***^ | 3.37^***^ | 4.74^***^ | 5.41^***^ | 4.67^***^ | 0.30^NS^ | 0.38^NS^ | ---- | 0.47^NS^ |
| FN13 | 4.24^***^ | 2.21^***^ | 1.21^*4.75**^ | 0.84^NS^ | 3.86^***^ | 4.58^***^ | 5.96^***^ | 4.20^***^ | 3.45^***^ | 0.92^NS^ | 1.60^**^ | 1.22^*^ | ---- |
| FN14 | 4.69^***^ | 2.66^***^ | 0.76^NS^ | 1.29^*^ | 3.41^***^ | 5.03^***^ | 6.41^***^ | 3.74^***^ | 3.00^***^ | 1.37^*^ | 2.05^***^ | 1.67^**^ | 0.45^NS^ |
| FN15 | 10.19^***^ | 8.16^***^ | 4.75^***^ | 6.79^***^ | 2.09^***^ | 10.54^***^ | 11.91^***^ | 1.76^**^ | 2.50^***^ | 6.88^***^ | 7.55^***^ | 7.17^***^ | 5.96^***^ |
| FN16 | 6.76^***^ | 4.73^***^ | 1.31^*^ | 3.36^***^ | 1.34^*^ | 7.10^***^ | 8.48^***^ | 1.68^**^ | 0.93^NS^ | 3.44^***^ | 4.12^***^ | 3.74^***^ | 2.52^***^ |
| FN17 | 2.80^***^ | 0.77^NS^ | 2.64^***^ | 0.59^NS^ | 5.30^***^ | 3.15^***^ | 4.52^***^ | 5.63^***^ | 4.88^***^ | 0.51^NS^ | 0.17^NS^ | 0.22^NS^ | 1.43^*^ |
| FN18 | 6.17^***^ | 8.20^***^ | 11.62^***^ | 9.57^***^ | 14.27^***^ | 5.83^***^ | 4.45^***^ | 14.61^***^ | 13.86^***^ | 9.49^***^ | 8.81^***^ | 9.19^***^ | 10.41^***^ |
| FN19 | 1.97^***^ | 0.04^NS^ | 3.46^***^ | 1.41^*^ | 6.11^***^ | 2.33^***^ | 3.71^***^ | 6.45^***^ | 5.70^***^ | 1.33^*^ | 0.65^NS^ | 1.03^NS^ | 2.25^***^ |
| FN20 | 2.76^***^ | 4.79^***^ | 8.21^***^ | 6.16^***^ | 10.87^***^ | 2.42^***^ | 1.04^NS^ | 11.20^***^ | 10.45^***^ | 6.08^***^ | 5.40^***^ | 5.78^***^ | 7.00^***^ |
| FN21 | 3.52^***^ | 5.55^***^ | 8.96^***^ | 6.92^***^ | 11.62^***^ | 3.17^***^ | 1.80^**^ | 11.95^***^ | 11.21^***^ | 6.83^***^ | 6.16^***^ | 6.54^***^ | 7.75^***^ |
| FN22 | 0.47^NS^ | 1.56^**^ | 4.98^***^ | 2.93^***^ | 7.63^***^ | 0.82^NS^ | 2.19^***^ | 7.97^***^ | 7.22^***^ | 2.85^***^ | 2.17^***^ | 2.55^***^ | 3.77^***^ |
| FN23 | 1.80^**^ | 0.23^NS^ | 3.64^***^ | 1.60^**^ | 6.30^***^ | 2.15^***^ | 3.52^***^ | 6.63^***^ | 5.89^***^ | 1.51^**^ | 0.84^NS^ | 1.22^*^ | 2.43^***^ |
| FN24 | 0.77^NS^ | 1.25^*^ | 4.67^***^ | 2.62^***^ | 7.32^***^ | 1.12^*^ | 2.49^***^ | 7.66^***^ | 6.91^***^ | 2.54^***^ | 1.86^***^ | 2.24^***^ | 3.46^***^ |
| FN25 | 0.78^NS^ | 1.24^*^ | 4.66^***^ | 2.61^***^ | 7.31^***^ | 1.13^*^ | 2.50^***^ | 7.65^***^ | 6.90^***^ | 2.53^***^ | 1.85^***^ | 2.23^***^ | 3.45^***^ |

NS *p* > 0.05, * *p* ≤ 0.05, ** *p* ≤ 0.01, *** *p* ≤ 0.001

**Table S5.** (continued)

| Tree | FN14 | FN15 | FN16 | FN17 | FN18 | FN19 | FN20 | FN21 | FN22 | FN23 | FN24 | FN25 |
| --- | --- | --- | --- | --- | --- | --- | --- | --- | --- | --- | --- | --- |
| FN01 | 3.45^***^ | 5.93^***^ | 3.52^***^ | 1.00^*^ | 7.50^***^ | 1.46^**^ | 5.75^***^ | 4.82^***^ | 0.48^NS^ | 0.98^*^ | 0.32^**^ | 0.12 |
| FN02 | 1.92^***^ | 4.40^***^ | 1.99^***^ | 0.53^NS^ | 9.03^***^ | 0.07^NS^ | 7.29^***^ | 6.35^***^ | 1.05^*^ | 0.54^NS^ | 1.85^***^ | 1.65^***^ |
| FN03 | 2.37^***^ | 0.10^NS^ | 2.31^***^ | 4.83^***^ | 13.33^***^ | 4.37^***^ | 11.59^***^ | 10.65^***^ | 5.35^***^ | 4.85^***^ | 6.15^***^ | 5.95^***^ |
| FN04 | 1.29^**^ | 1.18^*^ | 1.23^**^ | 3.74^***^ | 12.24^***^ | 3.29^***^ | 10.50^***^ | 9.56^***^ | 4.27^***^ | 3.76^***^ | 5.06^***^ | 4.87^***^ |
| FN05 | 1.17^*^ | 1.30^**^ | 1.11^*^ | 3.62^***^ | 12.13^***^ | 3.17^***^ | 10.38^***^ | 9.44^***^ | 4.15^***^ | 3.64^***^ | 4.94^***^ | 4.75^***^ |
| FN06 | 3.33^***^ | 5.80^***^ | 3.40^***^ | 0.88^NS^ | 7.62^***^ | 1.33^**^ | 5.88^***^ | 4.94^***^ | 0.35^NS^ | 0.86^NS^ | 0.44^NS^ | 0.24^NS^ |
| FN07 | 4.69^***^ | 7.17^***^ | 4.76^***^ | 2.24^***^ | 6.26^***^ | 2.70^***^ | 4.51^***^ | 3.58^***^ | 1.72^***^ | 2.22^***^ | 0.92^*^ | 1.12^*^ |
| FN08 | 1.63^***^ | 0.84^NS^ | 1.56^***^ | 4.08^***^ | 12.58^***^ | 3.62^***^ | 10.84^***^ | 9.90^***^ | 4.60^***^ | 4.10^***^ | 5.40^***^ | 5.20^***^ |
| FN09 | 1.31^**^ | 1.17^*^ | 1.23^**^ | 3.76^***^ | 12.26^***^ | 3.30^***^ | 10.51^***^ | 9.58^***^ | 4.28^***^ | 3.78^***^ | 5.08^***^ | 4.88^***^ |
| FN10 | 0.56^NS^ | 3.03^***^ | 0.63^NS^ | 1.89^***^ | 10.39^***^ | 1.43^**^ | 8.65^***^ | 7.71^***^ | 2.41^***^ | 1.91^***^ | 3.21^***^ | 3.01^***^ |
| FN11 | 2.62^***^ | 5.10^***^ | 2.69^***^ | 0.17^NS^ | 8.33^***^ | 0.63^NS^ | 6.58^***^ | 5.65^***^ | 0.35^NS^ | 0.15^NS^ | 1.15^*^ | 0.95^*^ |
| FN12 | 1.55^***^ | 4.03^***^ | 1.62^***^ | 0.90^NS^ | 9.40^***^ | 0.44^NS^ | 7.65^***^ | 6.72^***^ | 1.42^**^ | 0.91^*^ | 2.22^***^ | 2.02^***^ |
| FN13 | 1.09^*^ | 3.56^***^ | 1.15^*^ | 1.36^**^ | 9.87^***^ | 0.91^NS^ | 8.12^***^ | 7.18^***^ | 1.89^***^ | 1.38^**^ | 2.68^***^ | 2.49^***^ |
| FN14 | ---- | 2.47^***^ | 0.07^NS^ | 2.45^***^ | 10.95^***^ | 1.99^***^ | 9.21^***^ | 8.27^***^ | 2.97^***^ | 2.47^***^ | 3.77^***^ | 3.57^***^ |
| FN15 | 5.51^***^ | ---- | 2.41^***^ | 4.92^***^ | 13.43^***^ | 4.47^***^ | 11.68^***^ | 10.74^***^ | 5.45^***^ | 4.94^***^ | 8.24^***^ | 6.05^***^ |
| FN16 | 2.07^***^ | 3.44^***^ | ---- | 2.52^***^ | 11.02^***^ | 2.06^***^ | 9.28^***^ | 8.34^***^ | 3.04^***^ | 2.54^***^ | 3.84^***^ | 3.64^***^ |
| FN17 | 1.88^***^ | 7.39^***^ | 3.95^***^ | ---- | 8.50^***^ | 0.46^NS^ | 6.76^***^ | 5.82^***^ | 0.52^NS^ | 0.02^NS^ | 1.32^**^ | 1.12^*^ |
| FN18 | 10.86^***^ | 16.37^***^ | 12.93^***^ | 8.98^***^ | ---- | 8.96^***^ | 1.74^***^ | 2.68^***^ | 7.98^***^ | 8.48^***^ | 7.18^***^ | 7.38^***^ |
| FN19 | 2.70^***^ | 8.21^***^ | 4.77^***^ | 0.82^NS^ | 8.16^***^ | ---- | 7.21^***^ | 6.28^***^ | 0.98^*^ | 0.47^NS^ | 1.78^***^ | 1.58^***^ |
| FN20 | 7.45^***^ | 12.96^***^ | 9.52^***^ | 5.57^***^ | 3.41^***^ | 4.75^***^ | ---- | 0.94^*^ | 6.23^***^ | 6.74^***^ | 5.44^***^ | 5.64^***^ |
| FN21 | 8.20^***^ | 13.71^***^ | 10.27^***^ | 6.32^***^ | 2.66^***^ | 5.50^***^ | 0.75^NS^ | ---- | 5.30^***^ | 5.80^***^ | 4.50^***^ | 4.70^***^ |
| FN22 | 4.22^***^ | 9.72^***^ | 6.29^***^ | 2.34^***^ | 6.64^***^ | 1.52^**^ | 3.23^***^ | 3.99^***^ | ---- | 0.51^NS^ | 0.80^NS^ | 0.60^NS^ |
| FN23 | 2.88^***^ | 8.39^***^ | 4.95^***^ | 1.00^NS^ | 7.98^***^ | 0.18^NS^ | 4.57^***^ | 5.32^***^ | 1.33^*^ | ---- | 1.30^**^ | 1.10^*^ |
| FN24 | 3.91^***^ | 9.41^***^ | 5.98^***^ | 2.02^***^ | 6.95^***^ | 1.21^*^ | 3.54^***^ | 4.29^***^ | 0.31^NS^ | 1.02^NS^ | ---- | 0.20^NS^ |
| FN25 | 3.90^***^ | 9.40^***^ | 5.97^***^ | 2.02^***^ | 6.96^***^ | 1.20^*^ | 3.55^***^ | 4.30^***^ | 0.32^NS^ | 1.01^NS^ | 0.00^NS^ | ---- |

**Table S6.** Comparison of significant differences in mean stone transverse diameter (below the diagonal) and longitudinal diameter (above the diagonal) from individuals of *Malania oleifera* in Funing (LSD test)

| Tree | FN01 | FN02 | FN03 | FN04 | FN05 | FN06 | FN07 | FN08 | FN09 | FN10 | FN11 | FN12 | FN13 |
| --- | --- | --- | --- | --- | --- | --- | --- | --- | --- | --- | --- | --- | --- |
| FN01 | ---- | 0.94^*^ | 2.39^***^ | 0.78^NS^ | 0.66^NS^ | 2.16^***^ | 1.87^***^ | 2.69^***^ | 1.17^**^ | 0.96^*^ | 2.02^***^ | 0.52^NS^ | 0.92^*^ |
| FN02 | 0.92^**^ | ---- | 1.45^***^ | 0.16^NS^ | 1.60^***^ | 1.22^**^ | 2.81^***^ | 1.75^***^ | 0.77^NS^ | 0.02^NS^ | 1.08^*^ | 1.46^***^ | 1.85^***^ |
| FN03 | 3.05^***^ | 2.13^***^ | ---- | 1.61^***^ | 3.05^***^ | 0.24^NS^ | 4.26^***^ | 0.30^NS^ | 2.22^***^ | 1.43^***^ | 0.37^NS^ | 2.92^***^ | 3.31^***^ |
| FN04 | 0.85^*^ | 0.06^NS^ | 2.20^***^ | ---- | 1.44^***^ | 1.38^***^ | 2.65^***^ | 1.91^***^ | 0.61^NS^ | 0.18^NS^ | 1.24^**^ | 1.30^**^ | 1.70^***^ |
| FN05 | 2.15^***^ | 1.23^**^ | 0.90^*^ | 1.30^**^ | ---- | 2.82^***^ | 1.21^**^ | 3.35^***^ | 0.83^*^ | 1.62^***^ | 2.68^***^ | 0.14^NS^ | 0.26^NS^ |
| FN06 | 2.29^***^ | 1.37^***^ | 0.76^NS^ | 1.44^***^ | 0.14^NS^ | ---- | 4.03^***^ | 0.53^NS^ | 1.99^***^ | 1.19^**^ | 0.14^NS^ | 2.68^***^ | 3.07^***^ |
| FN07 | 0.71^NS^ | 1.63^***^ | 3.76^***^ | 1.56^***^ | 2.86^***^ | 3.00^***^ | ---- | 4.56^***^ | 2.04^***^ | 2.83^***^ | 3.89^***^ | 1.35^***^ | 0.95^*^ |
| FN08 | 5.75^***^ | 4.84^*^ | 2.70^***^ | 4.90^***^ | 3.60^***^ | 3.46^***^ | 6,46^***^ | ---- | 2.52^***^ | 1.73^***^ | 0.67^NS^ | 3.22^***^ | 3.61^***^ |
| FN09 | 3.41^***^ | 2.49^***^ | 0.36^NS^ | 2.56^***^ | 1.26^**^ | 1.12^**^ | 4.12^***^ | 2.35^***^ | ---- | 0.79^NS^ | 1.85^***^ | 0.69^NS^ | 1.08^**^ |
| FN10 | 1.11^**^ | 0.20^NS^ | 1.94^***^ | 0.26^NS^ | 1.04^*^ | 1.17^**^ | 1.82^***^ | 4.64^***^ | 2.29^***^ | ---- | 1.06^*^ | 1.48^***^ | 1.88^***^ |
| FN11 | 4.39^***^ | 3.47^***^ | 1.34^**^ | 3.54^***^ | 2.24^***^ | 2.10^***^ | 5.10^***^ | 1.36^***^ | 0.98^*^ | 3.27^***^ | ---- | 2.54^***^ | 2.93^***^ |
| FN12 | 1.11^**^ | 2.03^***^ | 4.16^***^ | 1.97^***^ | 3.26^***^ | 3.40^***^ | 0.40^NS^ | 6.87^***^ | 4.52^***^ | 2.23^***^ | 5.50^***^ | ---- | 0.39^NS^ |
| FN13 | 0.66^NS^ | 0.26^NS^ | 2.39^***^ | 0.19^NS^ | 1.49^***^ | 1.63^***^ | 1.37^***^ | 5.10^***^ | 2.75^***^ | 0.46^NS^ | 3.73^***^ | 1.77^**^ | ---- |
| FN14 | 1.23^**^ | 0.31^NS^ | 1.82^***^ | 0.38^NS^ | 0.92^*^ | 1.06^**^ | 1.94^***^ | 4.52^***^ | 2.18^***^ | 0.12^NS^ | 3.16^***^ | 2.34^***^ | 0.57^**^ |
| FN15 | 6.99^***^ | 6.00^***^ | 3.87^***^ | 6.07^***^ | 4.77^***^ | 4.63^***^ | 7.63^***^ | 1.17^**^ | 3.51^***^ | 5.81^***^ | 2.53^***^ | 8.03^***^ | 6.26^***^ |
| FN16 | 4.03^***^ | 3.12^***^ | 0.98^*^ | 3.18^***^ | 1.88^***^ | 1.74^***^ | 4.74^***^ | 1.72^**^ | 0.62^NS^ | 2.92^***^ | 0.36^NS^ | 5.15^***^ | 3.37^***^ |
| FN17 | 4.41^***^ | 3.49^***^ | 1.36^**^ | 3.56^***^ | 2.26^***^ | 2.12^***^ | 5.12^***^ | 1.35^**^ | 1.00^*^ | 3.29^***^ | 0.02^NS^ | 5.52^***^ | 3.75^***^ |
| FN18 | 2.21^***^ | 3.13^***^ | 5.26^***^ | 3.06^***^ | 4.36^***^ | 4.50^***^ | 1.50^***^ | 7.96^***^ | 5.62^***^ | 3.33^***^ | 6.60^***^ | 1.10^**^ | 2.87^***^ |
| FN19 | 1.12^**^ | 0.21^NS^ | 1.93^***^ | 0.27^NS^ | 1.03^*^ | 1.17^**^ | 1.83^***^ | 4.63^***^ | 2.28^***^ | 0.01^NS^ | 3.26^***^ | 2.34^***^ | 0.47^NS^ |
| FN20 | 2.75^***^ | 3.66^***^ | 5.80^***^ | 3.60^***^ | 4.90^***^ | 5.04^***^ | 2.04^***^ | 8.50^***^ | 6.15^***^ | 3.86^***^ | 7.14^***^ | 1.63^***^ | 3.40^***^ |
| FN21 | 1.05^*^ | 1.97^***^ | 4.10^***^ | 1.90^***^ | 3.20^***^ | 3.34^***^ | 0.34^NS^ | 6.81^***^ | 4.46^***^ | 2.17^***^ | 5.44^***^ | 0.06^NS^ | 1.71^***^ |
| FN22 | 0.72^NS^ | 0.20^NS^ | 2.33^***^ | 0.13^NS^ | 1.43^***^ | 1.57^***^ | 1.43^***^ | 5.03^***^ | 2.69^***^ | 0.39^NS^ | 3.67^***^ | 1.83^***^ | 0.06^NS^ |
| FN23 | 1.61^**^ | 0.70^NS^ | 1.43^***^ | 0.76^NS^ | 0.53^NS^ | 0.67^NS^ | 2.32^***^ | 4.14^***^ | 1.79^***^ | 0.50^NS^ | 2.77^***^ | 2.73^***^ | 0.96^*^ |
| FN24 | 0.46^NS^ | 0.46^NS^ | 2.59^***^ | 0.39^NS^ | 1.69^***^ | 1.83^***^ | 1.17^**^ | 5.29^***^ | 2.95^***^ | 0.65^NS^ | 3.93^***^ | 1.57^***^ | 0.20^NS^ |
| FN25 | 0.75^NS^ | 0.16^NS^ | 2.30^***^ | 0.10^NS^ | 1.40^***^ | 1.54^***^ | 1.46^***^ | 5.00^***^ | 2.66^***^ | 0.36^NS^ | 3.64^***^ | 1.87^***^ | 0.09^NS^ |

NS *p* > 0.05, * *p* ≤ 0.05, ** *p* ≤ 0.01, *** *p* ≤ 0.001

**Table S6.** (continued)

| Tree | FN14 | FN15 | FN16 | FN17 | FN18 | FN19 | FN20 | FN21 | FN22 | FN23 | FN24 | FN25 |
| --- | --- | --- | --- | --- | --- | --- | --- | --- | --- | --- | --- | --- |
| FN01 | 0.66^NS^ | 3.83^***^ | 2.55^***^ | 1.27^**^ | 6.24^***^ | 3.01^***^ | 6.96^***^ | 4.94^***^ | 1.74^***^ | 1.87^***^ | 3.32^***^ | 2.53^***^ |
| FN02 | 0.28^NS^ | 2.89^**^ | 1.61^***^ | 0.33^NS^ | 7.18^***^ | 3.94^***^ | 7.90^***^ | 5.88^***^ | 2.67^***^ | 2.81^***^ | 4.25^***^ | 3.47^***^ |
| FN03 | 1.74^***^ | 1.43^***^ | 0.16^NS^ | 1.13^**^ | 8.63^***^ | 5.40^***^ | 9.35^***^ | 7.33^***^ | 4.13^***^ | 4.26^***^ | 5.71^***^ | 4.92^***^ |
| FN04 | 0.12^NS^ | 3.05^***^ | 1.77^***^ | 0.49^NS^ | 7.02^***^ | 3.79^***^ | 7.74^***^ | 5.72^***^ | 2.52^***^ | 2.65^***^ | 4.10^***^ | 3.31^***^ |
| FN05 | 1.32^**^ | 4.49^***^ | 3.21^***^ | 1.93^***^ | 5.58^***^ | 2.35^***^ | 6.30^***^ | 4.28^***^ | 1.07^**^ | 1.21^**^ | 2.66^***^ | 1.87^***^ |
| FN06 | 1.50^***^ | 1.67^***^ | 0.39^NS^ | 0.89^*^ | 8.40^***^ | 5.16^***^ | 9.12^***^ | 7.10^***^ | 3.89^***^ | 4.03^***^ | 5.47^***^ | 4.69^***^ |
| FN07 | 2.53^***^ | 5.70^***^ | 4.42^***^ | 3.14^***^ | 4.37^***^ | 1.13^**^ | 5.09^***^ | 3.07^***^ | 0.14^NS^ | 0.00^NS^ | 1.45^***^ | 0.66^NS^ |
| FN08 | 2.04^***^ | 1.14^**^ | 0.14^NS^ | 1.43^***^ | 8.93^***^ | 5.70^***^ | 9.65^***^ | 7.63^***^ | 4.43^***^ | 4.56^***^ | 6.01^****^ | 5.22^***^ |
| FN09 | 0.49^NS^ | 3.66^***^ | 2.38^***^ | 1.10^**^ | 6.41^***^ | 3.17^***^ | 7.13^***^ | 5.11^***^ | 1.90^***^ | 2.04^***^ | 3.48^***^ | 2.70^***^ |
| FN10 | 0.31^NS^ | 2.86^***^ | 1.59^***^ | 0.30^NS^ | 7.20^***^ | 3.97^***^ | 7.92^***^ | 5.90^***^ | 2.70^***^ | 2.83^***^ | 4.28^***^ | 3.49^***^ |
| FN11 | 1.36^***^ | 1.81^***^ | 0.53^NS^ | 0.75^NS^ | 8.26^***^ | 5.02^***^ | 8.98^***^ | 6.96^***^ | 3.75^***^ | 3.89^***^ | 5.33^***^ | 4.55^***^ |
| FN12 | 1.18^**^ | 4.35^***^ | 3.07^***^ | 1.79^***^ | 5.72^***^ | 2.48^***^ | 6.44^***^ | 4.42^***^ | 1.21^**^ | 1.35^***^ | 2.79^*^ | 2.01^***^ |
| FN13 | 1.57^***^ | 4.74^***^ | 3.46^***^ | 2.18^***^ | 5.33^***^ | 2.09^***^ | 6.04^***^ | 4.03^***^ | 0.82^*^ | 0.95^*^ | 2.40^***^ | 1.62^***^ |
| FN14 | ---- | 3.17^***^ | 1.89^***^ | 0.61^NS^ | 6.90^***^ | 3.66^***^ | 7.62^***^ | 5.60^***^ | 2.39^***^ | 2.53^***^ | 3.97^***^ | 3.19^***^ |
| FN15 | 5.69^***^ | ---- | 1.28^**^ | 2.56^***^ | 10.07^***^ | 6.83^***^ | 10.79^***^ | 8.77^***^ | 5.56^***^ | 5.70^***^ | 7.14^***^ | 6.36^***^ |
| FN16 | 2.80^***^ | 2.89^***^ | ---- | 1.28^**^ | 8.79^***^ | 5.55^***^ | 9.51^***^ | 7.49^***^ | 4.28^***^ | 4.42^***^ | 5.86^***^ | 5.08^***^ |
| FN17 | 3.18^***^ | 2.51^***^ | 0.38^NS^ | ---- | 7.51^***^ | 4.27^***^ | 8.23^***^ | 6.21^***^ | 3.00^***^ | 3.14^***^ | 4.58^***^ | 3.80^***^ |
| FN18 | 3.44^***^ | 9.13^***^ | 6.24^***^ | 6.62^***^ | ---- | 3.24^***^ | 0.72^NS^ | 1.30^**^ | 4.51^***^ | 4.37^***^ | 2.93^***^ | 3.71^***^ |
| FN19 | 0.11^NS^ | 5.80^***^ | 2.91^***^ | 3.28^***^ | 3.33^***^ | ---- | 3.95^***^ | 1.94^***^ | 1.27^**^ | 1.14^**^ | 0.31^NS^ | 0.47^NS^ |
| FN20 | 3.98^***^ | 9.67^***^ | 6.78^***^ | 7.15^***^ | 0.54^NS^ | 3.87^***^ | ---- | 2.02^***^ | 5.23^***^ | 5.09^***^ | 3.64^***^ | 4.43^***^ |
| FN21 | 2.28^***^ | 7.97^***^ | 5.08^***^ | 5.46^***^ | 1.16^**^ | 2.18^***^ | 1.69^***^ | ---- | 3.21^***^ | 3.07^***^ | 1.63^***^ | 2.41^***^ |
| FN22 | 0.51^NS^ | 6.20^***^ | 3.31^***^ | 3.69^***^ | 2.93^***^ | 0.40^NS^ | 3.47^***^ | 1.77^***^ | ---- | 0.13^NS^ | 1.58^***^ | 0.80^NS^ |
| FN23 | 0.39^NS^ | 5.30^***^ | 2.42^***^ | 2.79^***^ | 3.83^***^ | 0.49^NS^ | 4.36^***^ | 2.67^***^ | 0.90^*^ | ---- | 1.44^***^ | 0.66^NS^ |
| FN24 | 0.77^NS^ | 6.46^***^ | 3.57^***^ | 3.95^***^ | 2.67^***^ | 0.66^NS^ | 3.21^***^ | 1.51^***^ | 0.26^NS*^ | 1.16^**^ | ---- | 0.78^NS^ |
| FN25 | 0.48^NS^ | 6.17^***^ | 3.28^***^ | 3.65^***^ | 2.96^***^ | 0.37^NS^ | 3.50^***^ | 1.80^***^ | 0.03^NS^ | 0.86^*^ | 0.29^NS^ | ---- |

**Table S7.** Comparison of significant differences in seed oil content (%) from individuals of *Malania oleifera* in Guangnan (LSD test)

| Tree | GN01 | GN02 | GN03 | GN04 | GN05 | GN06 | GN07 | GN08 | GN09 | GN10 | GN11 | GN12 | GN13 | GN14 |
| --- | --- | --- | --- | --- | --- | --- | --- | --- | --- | --- | --- | --- | --- | --- |
| GN01 | ---- |  |  |  |  |  |  |  |  |  |  |  |  |  |
| GN02 | 4.66^**^ | ---- |  |  |  |  |  |  |  |  |  |  |  |  |
| GN03 | 0.98^NS^ | 5.64^***^ | ---- |  |  |  |  |  |  |  |  |  |  |  |
| GN04 | 0.50^NS^ | 4.16^*^ | 1.48^NS^ | ---- |  |  |  |  |  |  |  |  |  |  |
| GN05 | 2.48^NS^ | 2.17^NS^ | 3.47^*^ | 1.99^NS^ | ---- |  |  |  |  |  |  |  |  |  |
| GN06 | 1.68^NS^ | 3.49^*^ | 2.15^NS^ | 0.66^NS^ | 1.31^NS^ | ---- |  |  |  |  |  |  |  |  |
| GN07 | 9.12^***^ | 4.47^**^ | 10.10^***^ | 8.62^***^ | 6.64^***^ | 7.95^**^ | ---- |  |  |  |  |  |  |  |
| GN08 | 0.95^NS^ | 3.70^*^ | 1.93^NS^ | 0.45^NS^ | 1.53^NS^ | 0.22^NS^ | 8.17^***^ | ---- |  |  |  |  |  |  |
| GN09 | 1.63^NS^ | 6.30^***^ | 0.66^NS^ | 2.14^NS^ | 4.13^*^ | 2.81^NS^ | 10.76^***^ | 2.59^NS^ | ---- |  |  |  |  |  |
| GN10 | 0.66^NS^ | 4.00^*^ | 1.64^NS^ | 0.16^NS^ | 1.83^NS^ | 0.51^NS^ | 8.47^***^ | 0.30^NS^ | 2.30^NS^ | ---- |  |  |  |  |
| GN11 | 1.93^NS^ | 2.72^NS^ | 2.91^NS^ | 1.43^NS^ | 0.56^NS^ | 0.76^**^ | 7.19^***^ | 0.98^NS^ | 3.57^*^ | 1.27^NS^ | ---- |  |  |  |
| GN12 | 0.53^NS^ | 4.12^*^ | 1.51^NS^ | 0.03^NS^ | 1.96^NS^ | 0.64^NS^ | 8.59^***^ | 0.42^NS^ | 2.17^NS^ | 0.13^NS^ | 1.40^NS^ | ---- |  |  |
| GN13 | 6.02^***^ | 10.68^***^ | 5.04^**^ | 6.52^***^ | 8.51^***^ | 7.19^**^ | 15.15^***^ | 6.98^***^ | 4.38^**^ | 6.68^***^ | 7.95^***^ | 6.55^***^ | ---- |  |
| GN14 | 4.19^*^ | 8.85^***^ | 3.21^*^ | 4.69^**^ | 6.68^***^ | 5.36^***^ | 13.32^***^ | 5.14^**^ | 2.55^NS^ | 4.85^**^ | 6.12^***^ | 4.72^**^ | 1.83^NS^ | ---- |
| GN15 | 1.04^NS^ | 5.70^***^ | 0.06^NS^ | 1.54^NS^ | 3.53^*^ | 2.21^NS^ | 10.17^***^ | 2.00^NS^ | 0.60^NS^ | 1.70^NS^ | 2.97^NS^ | 1.57^NS^ | 4.98^**^ | 3.15^NS^ |
| GN16 | 3.29^*^ | 7.95^***^ | 2.31^NS^ | 3.79^*^ | 5.78^***^ | 4.46^**^ | 12.41^***^ | 4.24^**^ | 1.65^NS^ | 3.94^*^ | 5.22^**^ | 3.82^*^ | 2.73^NS^ | 0.90^NS^ |
| GN17 | 0.72^NS^ | 5.37^***^ | 0.26^NS^ | 1.22^NS^ | 3.20^*^ | 1.86^NS^ | 9.84^***^ | 1.67^NS^ | 0.92^NS^ | 1.37^NS^ | 2.64^NS^ | 1.25^NS^ | 5.31^**^ | 3.47^*^ |
| GN18 | 5.70^***^ | 10.36^***^ | 4.72^**^ | 6.20^***^ | 8.19^***^ | 6.87^***^ | 14.82^***^ | 6.65^***^ | 4.06^*^ | 6.36^***^ | 7.63^***^ | 6.23^***^ | 0.32^NS^ | 1.51^NS^ |
| GN19 | 4.40^**^ | 9.05^***^ | 3.42^*^ | 4.90^**^ | 6.88^***^ | 5.56^***^ | 13.52^***^ | 5.35^**^ | 2.76^NS^ | 5.05^**^ | 6.33^***^ | 4.92^**^ | 1.63^NS^ | 0.20^NS^ |
| GN20 | 3.37^*^ | 8.03^***^ | 2.39^NS^ | 3.87^*^ | 5.86^***^ | 4.54^**^ | 12.50^***^ | 4.32^**^ | 1.73^NS^ | 4.03^*^ | 5.30^**^ | 3.90^*^ | 2.65^NS^ | 0.82^NS^ |
| GN21 | 1.48^NS^ | 6.14^***^ | 0.50^NS^ | 1.98^NS^ | 3.97^*^ | 2.65^NS^ | 10.61^***^ | 2.44^NS^ | 0.16^NS^ | 2.14^NS^ | 3.41^*^ | 2.01^NS^ | 4.54^**^ | 2.71^NS^ |
| GN22 | 1.08^NS^ | 5.74^***^ | 0.10^NS^ | 1.58^NS^ | 3.57^*^ | 2.25^NS^ | 10.20^***^ | 2.03^NS^ | 0.56^NS^ | 1.74^NS^ | 3.01^NS^ | 1.61^NS^ | 4.94^**^ | 3.11^NS^ |
| GN23 | 3.96^*^ | 8.61^***^ | 2.98^NS^ | 4.46^**^ | 6.45^***^ | 5.13^**^ | 13.08^***^ | 4.91^**^ | 2.32^NS^ | 4.61^**^ | 5.89^***^ | 4.49^NS^ | 2.07^NS^ | 0.23^NS^ |
| GN24 | 1.08^NS^ | 3.57^*^ | 2.06^NS^ | 0.58^NS^ | 1.40^NS^ | 0.08^NS^ | 8.04^***^ | 0.13^NS^ | 2.72^NS^ | 0.43^NS^ | 0.84^NS^ | 0.55^NS^ | 7.11^***^ | 5.28^**^ |
| GN25 | 1.72^NS^ | 6.37^***^ | 0.74^NS^ | 2.22^NS^ | 4.20^*^ | 2.88^NS^ | 10.84^***^ | 2.67^NS^ | 0.08^NS^ | 2.37^NS^ | 3.65^*^ | 2.25^NS^ | 4.31^**^ | 2.48^NS^ |
| GN26 | 2.95^NS^ | 1.70^NS^ | 3.93^*^ | 2.45^NS^ | 0.47^NS^ | 1.78^NS^ | 6.17^***^ | 2.00^NS^ | 4.59^**^ | 2.30^NS^ | 1.02^NS^ | 2.42^NS^ | 8.98^***^ | 7.14^**^ |
| GN27 | 7.10^***^ | 11.75^***^ | 6.12^***^ | 7.60^***^ | 9.59^***^ | 8.27^***^ | 16.22^***^ | 8.05^***^ | 5.46^***^ | 7.75^**^ | 9.03^***^ | 7.63^***^ | 1.07^NS^ | 2.91^NS^ |
| GN28 | 0.62^NS^ | 4.04^*^ | 1.60^NS^ | 0.12^**^ | 1.87^NS^ | 0.55^NS^ | 8.51^***^ | 0.34^NS^ | 2.25^NS^ | 0.04^NS^ | 1.31^NS^ | 0.09^NS^ | 6.64^***^ | 4.81^**^ |

NS *p* > 0.05, * *p* ≤ 0.05, ** *p* ≤ 0.01, *** *p* ≤ 0.001

**Table S7.** (continued)

| Tree | GN15 | GN16 | GN17 | GN18 | GN19 | GN20 | GN21 | GN22 | GN23 | GN24 | GN25 | GN26 | GN27 | GN28 |
| --- | --- | --- | --- | --- | --- | --- | --- | --- | --- | --- | --- | --- | --- | --- |
| GN01 |  |  |  |  |  |  |  |  |  |  |  |  |  |  |
| GN02 |  |  |  |  |  |  |  |  |  |  |  |  |  |  |
| GN03 |  |  |  |  |  |  |  |  |  |  |  |  |  |  |
| GN04 |  |  |  |  |  |  |  |  |  |  |  |  |  |  |
| GN05 |  |  |  |  |  |  |  |  |  |  |  |  |  |  |
| GN06 |  |  |  |  |  |  |  |  |  |  |  |  |  |  |
| GN07 |  |  |  |  |  |  |  |  |  |  |  |  |  |  |
| GN08 |  |  |  |  |  |  |  |  |  |  |  |  |  |  |
| GN09 |  |  |  |  |  |  |  |  |  |  |  |  |  |  |
| GN10 |  |  |  |  |  |  |  |  |  |  |  |  |  |  |
| GN11 |  |  |  |  |  |  |  |  |  |  |  |  |  |  |
| GN12 |  |  |  |  |  |  |  |  |  |  |  |  |  |  |
| GN13 |  |  |  |  |  |  |  |  |  |  |  |  |  |  |
| GN14 |  |  |  |  |  |  |  |  |  |  |  |  |  |  |
| GN15 | ---- |  |  |  |  |  |  |  |  |  |  |  |  |  |
| GN16 | 2.25^NS^ | ---- |  |  |  |  |  |  |  |  |  |  |  |  |
| GN17 | 0.32^NS^ | 2.57^NS^ | ---- |  |  |  |  |  |  |  |  |  |  |  |
| GN18 | 4.66^**^ | 2.41^NS^ | 4.98^**^ | ---- |  |  |  |  |  |  |  |  |  |  |
| GN19 | 3.35^*^ | 1.11^NS^ | 3.68^*^ | 1.31^NS^ | ---- |  |  |  |  |  |  |  |  |  |
| GN20 | 2.33^NS^ | 0.08^NS^ | 2.65^NS^ | 2.33^NS^ | 1.02^NS^ | ---- |  |  |  |  |  |  |  |  |
| GN21 | 0.44^NS^ | 1.81^NS^ | 0.77^NS^ | 4.22^*^ | 2.91^NS^ | 1.89^NS^ | ---- |  |  |  |  |  |  |  |
| GN22 | 0.04^NS^ | 2.21^NS^ | 0.36^NS^ | 4.62^**^ | 3.32^*^ | 2.29^NS^ | 0.40^NS^ | ---- |  |  |  |  |  |  |
| GN23 | 2.92^NS^ | 0.67^NS^ | 3.24^*^ | 1.74^NS^ | 0.44^NS^ | 0.59^NS^ | 2.47^NS^ | 2.88^NS^ | ---- |  |  |  |  |  |
| GN24 | 2.13^NS^ | 4.37^**^ | 1.80^NS^ | 6.79^***^ | 5.48^***^ | 4.46^**^ | 2.57^NS^ | 2.16^NS^ | 5.04^**^ | ---- |  |  |  |  |
| GN25 | 0.67^NS^ | 1.57^NS^ | 1.00^NS^ | 3.98^*^ | 2.68^NS^ | 1.66^NS^ | 0.23^NS^ | 0.64^NS^ | 2.24^NS^ | 2.80^NS^ | ---- |  |  |  |
| GN26 | 3.99^*^ | 6.24^***^ | 3.69^*^ | 8.65^***^ | 7.35^***^ | 6.32^***^ | 4.44^**^ | 4.03^**^ | 6.91^***^ | 1.87^NS^ | 4.67^**^ | ---- |  |  |
| GN27 | 6.06^***^ | 3.81^*^ | 6.38^***^ | 1.40^NS^ | 2.70^NS^ | 3.73^*^ | 5.61^***^ | 6.02^***^ | 3.14^NS^ | 8.18^***^ | 5.38^***^ | 10.05^***^ | ---- |  |
| GN28 | 1.66^NS^ | 3.90^*^ | 1.34^NS^ | 6.32^***^ | 5.01^**^ | 3.99^*^ | 2.10^NS^ | 1.70^NS^ | 4.57^**^ | 0.46^NS^ | 2.33^NS^ | 2.34^NS^ | 7.71^***^ | ---- |

**Table S8.** Comparison of significant differences in seed oil content (%) from individuals of *Malania oleifera* in Funing (LSD test)

| Tree | FN01 | FN02 | FN03 | FN04 | FN05 | FN06 | FN07 | FN08 | FN09 | FN10 | FN11 | FN12 | FN13 |
| --- | --- | --- | --- | --- | --- | --- | --- | --- | --- | --- | --- | --- | --- |
| FN01 | ---- |  |  |  |  |  |  |  |  |  |  |  |  |
| FN02 | 4.57^*^ | ---- |  |  |  |  |  |  |  |  |  |  |  |
| FN03 | 0.49^NS^ | 4.08^*^ | ---- |  |  |  |  |  |  |  |  |  |  |
| FN04 | 1.03^NS^ | 5.60^**^ | 1.52^NS^ | ---- |  |  |  |  |  |  |  |  |  |
| FN05 | 2.24^NS^ | 2.33^NS^ | 1.75^NS^ | 3.27^NS^ | ---- |  |  |  |  |  |  |  |  |
| FN06 | 0.19^NS^ | 4.76^**^ | 0.68^NS^ | 0.84^NS^ | 2.43^NS^ | ---- |  |  |  |  |  |  |  |
| FN07 | 0.13^NS^ | 4.70^*^ | 0.63^NS^ | 0.90^NS^ | 2.37^NS^ | 0.06^NS^ | ---- |  |  |  |  |  |  |
| FN08 | 0.13^NS^ | 4.44^*^ | 0.36^NS^ | 1.16^NS^ | 2.11^NS^ | 0.32^NS^ | 0.26^NS^ | ---- |  |  |  |  |  |
| FN09 | 1.71^NS^ | 6.29^***^ | 2.20^NS^ | 0.68^NS^ | 3.95^*^ | 1.52^NS^ | 1.58^NS^ | 1.84^NS^ | ---- |  |  |  |  |
| FN10 | 7.17^***^ | 2.60^NS^ | 6.68^***^ | 8.20^***^ | 4.93^**^ | 7.36^***^ | 7.30^***^ | 7.04^***^ | 8.88^***^ | ---- |  |  |  |
| FN11 | 1.59^NS^ | 6.16^***^ | 2.08^NS^ | 0.56^NS^ | 3.83^*^ | 1.40^NS^ | 1.46^NS^ | 1.72^NS^ | 0.12^NS^ | 8.76^***^ | ---- |  |  |
| FN12 | 2.37^NS^ | 2.20^NS^ | 1.88^NS^ | 3.40^NS^ | 0.13^NS^ | 2.56^NS^ | 2.50^NS^ | 2.24^NS^ | 4.08^*^ | 4.80^**^ | 3.96^*^ | ---- |  |
| FN13 | 0.17^NS^ | 4.74^*^ | 0.66^NS^ | 0.86^NS^ | 2.41^NS^ | 0.02^NS^ | 0.03^NS^ | 0.30^NS^ | 1.54^NS^ | 7.34^***^ | 1.42^NS^ | 2.53^NS^ | ---- |
| FN14 | 5.83^**^ | 1.26^NS^ | 5.34^**^ | 6.86^***^ | 3.59^*^ | 6.02^**^ | 5.97^**^ | 5.70^**^ | 7.54^***^ | 1.34^NS^ | 7.42^***^ | 3.46^NS^ | 6.00^**^ |
| FN15 | 5.83^**^ | 0.81^NS^ | 4.89^**^ | 6.41^***^ | 3.14^NS^ | 5.57^**^ | 5.52^**^ | 5.25^**^ | 7.01^**^ | 1.79^NS^ | 6.97^***^ | 3.01^NS^ | 5.55^**^ |
| FN16 | 1.27^NS^ | 3.30^NS^ | 0.78^NS^ | 2.30^NS^ | 0.97^NS^ | 1.46^NS^ | 1.40^NS^ | 1.14^NS^ | 2.98^NS^ | 5.90^**^ | 2.86^NS^ | 1.10^NS^ | 1.44^NS^ |
| FN17 | 0.44^NS^ | 4.13^*^ | 0.05^NS^ | 1.47^NS^ | 1.80^NS^ | 0.63^NS^ | 0.58^NS^ | 0.31^NS^ | 2.15^NS^ | 6.73^***^ | 2.03^NS^ | 1.93^NS^ | 0.61^NS^ |
| FN18 | 3.26^NS^ | 1.31^NS^ | 2.77^NS^ | 4.29^*^ | 1.02^NS^ | 3.45^NS^ | 3.40^NS^ | 3.13^NS^ | 4.97^**^ | 3.91^*^ | 4.85^**^ | 0.89^NS^ | 3.43^NS^ |
| FN19 | 2.16^NS^ | 6.73^**^ | 2.65^NS^ | 1.13^NS^ | 4.40^*^ | 1.97^NS^ | 2.20^NS^ | 2.29^NS^ | 0.45^NS^ | 9.33^***^ | 0.57^NS^ | 4.53^*^ | 1.99^NS^ |
| FN20 | 6.66^***^ | 2.09^NS^ | 6.17^***^ | 7.69^***^ | 4.42^*^ | 6.85^***^ | 6.79^***^ | 6.53^***^ | 8.37^**^ | 0.51^NS^ | 8.25^***^ | 4.29^*^ | 6.83^***^ |
| FN21 | 1.35^NS^ | 5.92^**^ | 1.84^NS^ | 0.32^**^ | 3.59^*^ | 1.16^NS^ | 1.22^NS^ | 1.48^NS^ | 0.36^NS^ | 8.52^***^ | 0.24^NS^ | 3.72^*^ | 1.18^NS^ |
| FN22 | 5.07^NS^ | 0.50^NS^ | 4.58^*^ | 6.10^***^ | 2.84^NS^ | 5.26^NS^ | 5.21^**^ | 4.94^**^ | 6.78^***^ | 2.10^NS^ | 6.66^***^ | 2.70^NS^ | 5.24^**^ |
| FN23 | 1.65^NS^ | 6.22^***^ | 2.15^NS^ | 0.62^NS^ | 3.89^*^ | 1.46^NS^ | 1.52^NS^ | 1.78^NS^ | 0.05^NS^ | 8.82^***^ | 0.06^NS^ | 4.02^*^ | 1.49^NS^ |
| FN24 | 0.20^NS^ | 4.37^*^ | 0.29^NS^ | 1.23^NS^ | 2.04^NS^ | 0.39^NS^ | 0.34^NS^ | 0.07^NS^ | 1.91^NS^ | 6.97^***^ | 1.79^NS^ | 2.17^NS^ | 0.37^NS^ |
| FN25 | 5.09^**^ | 0.52^NS^ | 4.69^*^ | 6.12^***^ | 2.85^NS^ | 5.28^**^ | 5.23^**^ | 4.96^**^ | 6.80^***^ | 2.08^NS^ | 6.68^***^ | 2.72^NS^ | 5.26^**^ |

NS *p* > 0.05, * *p* ≤ 0.05, ** *p* ≤ 0.01, *** *p* ≤ 0.001

**Table S8.** (continued)

| Tree | FN14 | FN15 | FN16 | FN17 | FN18 | FN19 | FN20 | FN21 | FN22 | FN23 | FN24 | FN25 |
| --- | --- | --- | --- | --- | --- | --- | --- | --- | --- | --- | --- | --- |
| FN01 |  |  |  |  |  |  |  |  |  |  |  |  |
| FN02 |  |  |  |  |  |  |  |  |  |  |  |  |
| FN03 |  |  |  |  |  |  |  |  |  |  |  |  |
| FN04 |  |  |  |  |  |  |  |  |  |  |  |  |
| FN05 |  |  |  |  |  |  |  |  |  |  |  |  |
| FN06 |  |  |  |  |  |  |  |  |  |  |  |  |
| FN07 |  |  |  |  |  |  |  |  |  |  |  |  |
| FN08 |  |  |  |  |  |  |  |  |  |  |  |  |
| FN09 |  |  |  |  |  |  |  |  |  |  |  |  |
| FN10 |  |  |  |  |  |  |  |  |  |  |  |  |
| FN11 |  |  |  |  |  |  |  |  |  |  |  |  |
| FN12 |  |  |  |  |  |  |  |  |  |  |  |  |
| FN13 |  |  |  |  |  |  |  |  |  |  |  |  |
| FN14 | ---- |  |  |  |  |  |  |  |  |  |  |  |
| FN15 | 0.45^NS^ | ---- |  |  |  |  |  |  |  |  |  |  |
| FN16 | 4.56^*^ | 4.11^*^ | ---- |  |  |  |  |  |  |  |  |  |
| FN17 | 5.39^**^ | 4.94^**^ | 0.83^NS^ | ---- |  |  |  |  |  |  |  |  |
| FN18 | 2.57^NS^ | 2.12^NS^ | 1.99^NS^ | 2.82^NS^ | ---- |  |  |  |  |  |  |  |
| FN19 | 7.99^***^ | 7.54^***^ | 3.43^NS^ | 2.60^NS^ | 5.42^**^ | ---- |  |  |  |  |  |  |
| FN20 | 0.83^NS^ | 1.28^NS^ | 5.39^**^ | 6.22^***^ | 3.40^NS^ | 8.82^***^ | ---- |  |  |  |  |  |
| FN21 | 7.18^***^ | 6.73^***^ | 2.62^NS^ | 1.79^NS^ | 4.61^*^ | 0.81^NS^ | 8.01^***^ | ---- |  |  |  |  |
| FN22 | 0.76^NS^ | 0.31^NS^ | 3.80^*^ | 4.63^*^ | 1.81^NS^ | 7.23^***^ | 1.58^NS^ | 6.43^***^ | ---- |  |  |  |
| FN23 | 7.48^***^ | 7.03^***^ | 2.92^NS^ | 2.10^NS^ | 4.92^**^ | 0.51^NS^ | 8.31^***^ | 0.30^NS^ | 6.73^***^ | ---- |  |  |
| FN24 | 5.63^**^ | 5.18^**^ | 1.07^NS^ | 0.24^NS^ | 3.06^NS^ | 2.36^NS^ | 6.46^***^ | 1.55^NS^ | 4.87^**^ | 1.86^NS^ | ---- |  |
| FN25 | 0.74^NS^ | 0.29^NS^ | 3.82^*^ | 4.65^*^ | 1.83^NS^ | 7.25^***^ | 1.57^NS^ | 6.44^***^ | 0.02^NS^ | 6.74^***^ | 4.89^*^ | ---- |
